# Long-Term Health and Cost Outcomes of a 24-Week Multicomponent Frailty Intervention in Older Adults

**DOI:** 10.1001/jamanetworkopen.2025.43278

**Published:** 2025-11-12

**Authors:** Sunghwan Ji, Jihye Lim, Tai Joon An, Geonyeong Jang, Ji Yeon Baek, Kunhee Park, Ju Jin Jung, Seon-Hee Cheon, Jun-Pyo Myong, Yun-Hee Lee, Juhee Cho, Jin Lee, Hojoon Sohn, Il-Young Jang

**Affiliations:** 1 Division of Geriatrics, Department of Internal Medicine, Asan Medical Center, University of Ulsan College of Medicine, Seoul, Korea; 2 Department of Information Medicine, Asan Medical Center, University of Ulsan College of Medicine, Seoul, Korea; 3 Department of Digital Health, Samsung Advanced Institute for Health Sciences and Technology, Sungkyunkwan University, Seoul, Korea; 4 Division of Gastroenterology and Hepatology, Department of Internal Medicine, Yeouido St Mary’s Hospital, College of Medicine, The Catholic University of Korea, Seoul, Korea; 5 Division of Pulmonology and Critical Care Medicine, Department of Internal Medicine, Yeouido St Mary’s Hospital, College of Medicine, The Catholic University of Korea, Seoul, Korea; 6 PyeongChang Health Center and County Hospital, Gangwon-do, Korea; 7 Department of Occupational and Environmental Medicine, Seoul St Mary’s Hospital, College of Medicine, The Catholic University of Korea, Seoul, Korea; 8 Department of Occupational & Environmental Medicine, College of Medicine, The Catholic University of Korea, Seoul, Korea; 9 Department of Clinical Research Design and Evaluation, SAIHST, Sungkyunkwan University, Seoul, Korea; 10 Center for Clinical Epidemiology, Samsung Medical Center, Seoul, Korea; 11 Department of Mental Health, Johns Hopkins Bloomberg School of Public Health, Baltimore, Maryland; 12 Department of Preventive Medicine, Seoul National University College of Medicine, Seoul, Korea; 13 Department of Human Systems Medicine, Seoul National University College of Medicine, Seoul, Korea; 14 Institute of Health Policy and Management, Seoul National University, Seoul, Korea

## Abstract

**Importance:**

While multicomponent frailty interventions have demonstrated short-term effectiveness, evidence on long-term health and cost outcomes is limited.

**Objective:**

To evaluate the 66-month health and cost outcomes of a multicomponent frailty intervention for older adults in rural Korea.

**Design, Setting, and Participants:**

This economic evaluation used data from the Aging Study of Pyeongchang Rural Area–Intervention Study, a nonrandomized clinical trial conducted from August 2015 to January 2017. Individual-level data were linked to National Health Insurance Service claims data through December 2021, and analyses were performed from June 2024 to August 2025. Participants were socioeconomically vulnerable adults aged 65 years and older living alone or receiving medical aid in a rural Korean community. Volunteers were enrolled in the intervention, while those who declined enrollment served as controls. Propensity score matching was performed using frailty, functional status, demographics, comorbidities, depression, and prior health service use.

**Intervention:**

A 24-week multicomponent program including structured exercise and nutrition, with depression management, deprescribing, and home hazard reduction provided by risk profile.

**Main Outcomes and Measures:**

Survival analyses assessed a composite of all-cause mortality or long-term care insurance eligibility over 66 months. Cost outcomes included cumulative health service–use costs and cost:benefit ratios, calculated as incremental costs relative to intervention costs.

**Results:**

Among 383 eligible adults, 187 volunteered to participate and 196 individuals served as controls; NHIS linkage was successful for 181 (96.8%) and 192 (98.0%) participants, respectively. Among 119 matched pairs (238 [68.9%] aged ≥75 years; 177 [74.4%] female), participants had longer composite outcome-free survival at 66 months (mean difference, 6.53 [95% CI, 1.38-11.68] months). Health service–use costs were consistently lower in the participants group, mainly from reduced hospitalizations and long-term care use, with per-person savings of $7688 (95% CI, 1197-14 615) at 66 months, representing a cost:benefit ratio of 8.82.

**Conclusions and Relevance:**

In this economic evaluation of a nonrandomized clinical trial, participants in a 24-week multicomponent intervention had longer survival free from death or long-term care eligibility and had lower health service–use costs over 66 months. These findings support consideration of broader implementation of community-based frailty interventions in older adults.

## Key Points

**Question:**

What are the long-term health and cost outcomes associated with a 24-week multicomponent frailty intervention for socioeconomically vulnerable older adults in rural Korea?

**Findings:**

In this economic evaluation of a nonrandomized trial of 119 matched pairs of older adults, participants had significantly longer survival free from death or long-term care eligibility and significantly lower cumulative health service-use costs ($7688 per person) over 66 months, yielding a cost:benefit ratio of 8.82.

**Meaning:**

This economic evaluation found that the 24-week multicomponent intervention was associated with sustained health and cost benefits in older adults, supporting broader implementation.

## Teaser

This economic evaluation examines health and cost outcomes of a 24-week multicomponent frailty intervention for older adults in rural Korea at 30 and 66 months.

## Introduction

Frailty, a multidimensional syndrome commonly associated with aging, increases risk of adverse health outcomes and is associated with increasing health care costs, emerging as a global health burden.^1^ Since frailty is a systemic syndrome, interventions targeting multiple physiological systems, such as exercise programs and nutritional support, have demonstrated efficacy.^2^ Systematic reviews show that multicomponent interventions integrating these approaches significantly reduce frailty.^3^ While their specific protocols differ, they share core principles, including a holistic assessment of needs, a comprehensive approach emphasizing physical activity and nutrition, and the aim of maintaining independence. The World Health Organization’s Integrated Care for Older People framework exemplifies these principles,^4^ and several clinical guidelines incorporate them.^5,6^ Research has demonstrated that these multicomponent programs can reduce frailty^7,8^ and mobility disability risk^9^ and improve well-being in older adults.^10^

However, current evidence on these interventions is largely limited to short-term outcomes, with follow-up periods typically limited to 1 year,^7,8,10^ or at most, 2 to 3 years.^9,11^ Economic evaluations are even more scarce: most are limited to 1 year^12,13,14^ to 3 years,^15^ with heterogeneous conclusions. While some multicomponent interventions, including exercise, nutrition counseling, and social support, have demonstrated potential economic benefits by reducing frailty progression^12,15^ and unplanned hospital admissions,^14^ others, such as primary care-based interventions, have not shown significant financial benefits.^13^ Likewise, key questions, such as the temporal patterns of intervention outcomes to determine when refresher programs are needed, as well as which populations should be prioritized, remain unanswered. These gaps underscore the need for further research to facilitate the implementation of such interventions.

Previously, our study team implemented a 24-week multicomponent frailty intervention program^16^—including group exercise, nutritional intervention, and a tailored combination of depression management, deprescribing, and home hazard reduction—for socioeconomically vulnerable (participants who were living alone or receiving medical aid designated for low-income status) older adults in a rural area of Korea. We found that by sustaining improvements in physical performance,^16^ the program was associated with reduced risk of disability progression,^17^ institutionalization, or death over 30 months.^18^ Building on these findings, this study leverages existing data of study participants, linked with National Health Insurance Service (NHIS) claims data. We compared participants and nonparticipants regarding survival free from death or long-term care insurance (LTCI) eligibility as a measure of independence and assessed cumulative health service-use costs with cost-benefit ratio.

## Methods

This economic evaluation was conducted as part of the Aging Study of Pyeongchang Rural Area–Intervention Study (ASPRA-IS), a prospective, single-group study implementing a 24-week multicomponent intervention for socioeconomically vulnerable adults aged 65 years and older in Pyeongchang County, Korea, conducted from August 2015 to January 2017.^16^ The intervention was approved by the institutional review board of Asan Medical Center and registered at ClinicalTrials.gov (NCT02554994); the nonparticipant comparison group was derived from the ASPRA observational cohort, also approved by the institutional review board of Asan Medical Center. All participants provided written informed consent. All participants of the current analysis (including intervention participants and intervention nonparticipants) provided written informed consent. This study is reported following the Strengthening the Reporting of Observational Studies in Epidemiology (STROBE) reporting guideline and the Consolidated Health Economic Evaluation Reporting Standards (CHEERS) reporting guideline.

All ASPRA-IS participants were recruited from the ASPRA cohort.^19^ ASPRA participants who were living alone or receiving medical aid designated for low-income status were invited to participate in the intervention program. A total of 1267 participants from ASPRA were screened, of whom 383 met the eligibility criteria. Among these, 187 participated in and adhered to the multicomponent intervention. A total of 196 individuals who were eligible but declined to participate served as the nonparticipants comparison group. Detailed eligibility criteria and study design are provided in eAppendix 1 and eFigure 1 in Supplement 1.

### Multicomponent Intervention Program

The 24-week multicomponent intervention program included group exercise, nutritional supplementation, depression management, deprescribing, and home hazard reduction, each tailored to meet the specific needs of participants (eAppendix 2 and eTable 1 in Supplement 1). All participants took part in the group exercise and nutritional program. Group exercise sessions were conducted for 60 minutes twice weekly.^20^ Nutritional supplementation included of 2 packs daily of a 125 mL liquid formula. Nutritional supplementation included 2 packs daily of a 125 mL liquid nutritional supplement (noncommercial formula prepared for the study; Maeil Dairies Co.).^21,22^

Participants who screened as having high risk of depression (CES-D score >20 points at baseline) were enrolled in the depression management program.^23^ Participants using 5 or more prescription medications at baseline participated in the deprescribing program.^24^ Finally, nurses and social workers assessed participants’ home environments using a checklist from the Centers for Disease Control and Prevention,^25^ and home hazards were mitigated through modifications.

The intervention was initiated 6 months after the baseline assessment and lasted for 6 months. After program completion, participants returned to usual care. The intervention was developed in collaboration with the Pyeongchang Health Center and County Hospital, together with community stakeholders, and the detailed protocol has been published previously.^16^ Meanwhile, the nonparticipant group continued to receive usual care.

### ASPRA-IS Cohort Data Linkage With the NHIS Database

For this study, data from the both groups were linked to the NHIS database using participant name, sex, and birth date, restricted to residents of Pyeongchang County. The NHIS is the universal health care system of Korea, providing comprehensive medical coverage. It also manages the LTCI program, which supports individuals requiring assistance with daily activities due to aging or disability. A detailed overview of NHIS and LTCI is available in eAppendix 3 in Supplement 1.

Among the initial 187 participants and 196 nonparticipants, 181 and 192 individuals, respectively, were successfully linked to the NHIS database (eFigure 2 in Supplement 1). The linked NHIS database provided data from 2012 to 2021, including medical service utilization and LTC use. Data on sensitive health conditions, such as psychiatric disorders, were excluded.

### Assessment of Intervention Costs

Total intervention costs included operational expenses (eg, coordinator salaries), direct intervention costs, and an estimated 20% for unmeasured components. For each participant, intervention component–specific costs were calculated based on individual adherence and summed to determine total cost per eligible participant. Detailed methods and adherence definitions are available in eAppendix 4 in Supplement 1. All costs are presented in US dollars, using a mean exchange rate of 1141 KR₩ per US $1 during the intervention period (2015-2017).

### Outcome Measures

Outcome measures were ascertained from the NHIS database, which ensured complete follow-up for all participants. We defined 2 key time points for outcome assessment: 30 months, aligned with previous studies,^17,18^ and 66 months after intervention initiation. Mortality data were obtained from Statistics Korea. LTCI eligibility was defined as receiving approval and a grade classification from the NHIS based on standardized assessment (eAppendix 3 in Supplement 1).

Health service use costs, including medical and LTC costs, included both out-of-pocket and NHIS-covered expenses. Considering that the analysis used claims data over 5 years, no discounting was applied. Lastly, the cost:benefit ratio was calculated by dividing the reduced health service–use costs in the participants group by the total intervention costs.

### Statistical Analysis

We performed 1:1 propensity score matching (PSM) using a nearest-neighbor method with a caliper width of 0.2 SD of the logit propensity score, including frailty index (eTable 2 in Supplement 1), gait speed, enrollment year, living alone, age group, chronic conditions, Cardiovascular Health Study frailty score, gender, medical aid status, prior-year medical costs, LTCI eligibility, and Center for Epidemiologic Studies Depression Scale (CES-D) score. We used standardized mean difference (SMD) to assess baseline balance. All subsequent analyses were conducted within the matched cohort.

For health outcome, Kaplan-Meier curves visualized the composite outcome of all-cause mortality and LTC eligibility over 66 months. Restricted mean survival time (RMST) and restricted mean time lost (RMTL) were estimated at 30 and 66 months, and between-group differences in RMST were assessed at these 2 time points.

For cost outcomes, we compared health service–use costs between groups using nonparametric paired bootstrap methods with 1000 replications to account for the skewed distribution of cost data, and between-group differences of cumulative costs were assessed at 30 and 66 months. Analyses were also stratified by medical and LTC expenditures. Details of the bootstrap analysis are in eAppendix 5 in Supplement 1.

All analyses were conducted using R software version 4.0.3 (R Project for Statistical Computing) between June 2024 and August 2025. Additional analyses, including multivariable-adjusted models and subgroup-specific analyses, inflation-adjusted analyses, *E*-value estimation, sensitivity analyses (alternative caliper widths and inverse probability weighting), and analyses by service type and principal diagnosis, are described in eAppendix 6 in Supplement 1. To correct for multiple comparisons, a 2-sided Bonferroni-adjusted significance level of *P* < .025 was applied to each of the 2 outcome types (health and cost), which were assessed at both 30 and 66 months.

## Results

### Baseline Characteristics

A total of 119 matched pairs (238 participants [68.9%] aged ≥75 years; 177 [74.4%] female) were included in the analysis. Before matching, the participants were generally older (SMD, 0.47), had higher male:female ratio (SMD, 0.47), lower grip strength (SMD, 0.42), more chronic conditions (SMD, 0.25), slower gait speed (SMD, 0.36), and greater frailty based on both the frailty phenotype (SMD, 0.30) and frailty index (SMD, 0.33) compared with nonparticipants. After PSM, the SMDs for age and number of chronic conditions were 0.17 and 0.13, respectively, and all other key variables had SMDs below 0.1 (Table). The unmatched participants were more frail and had lower physical function, while unmatched nonparticipants were healthier (younger, less frail, better physical function) than their matched counterparts (eTable 3 in Supplement 1). The distribution of propensity scores before and after matching is described in eFigure 3 in Supplement 1.

**Table.** Baseline Characteristics Before and After Propensity Score Matching

Characteristic	Before matching			After matching			
	Individuals, No. (%)		SMD	Individuals, No. (%)		SMD
	Participants (n = 181)	Nonparticipants (n = 192)		Participants (n = 119)	Nonparticipants (n = 119)	
Age, y						
65-69	13 (7.2)	39 (20.3)	0.47	12 (10.1)	10 (8.4)	0.17
70-74	36 (19.9)	45 (23.4)		25 (21.0)	27 (22.7)	
75-79	73 (40.3)	64 (33.3)		44 (37.0)	49 (41.2)	
80-84	45 (24.9)	27 (14.1)		28 (23.5)	21 (17.6)	
≥85	14 (7.7)	17 (8.9)		10 (8.4)	12 (10.1)	
Sex						
Female	141 (77.9)	129 (67.2)	0.47	90 (75.6)	87 (73.1)	0.06
Male	40 (22.1)	63 (32.8)		29 (24.4)	32 (26.9)	
Enrolled year						
2014	33 (18.2)	61 (31.8)	0.20	15 (12.6)	13 (10.9)	0.07
2015	82 (45.3)	63 (32.8)		98 (82.4)	102 (85.7)	
2016	66 (36.5)	68 (35.4)		6.04 (1.21)	5.98 (1.16)	
Medical aid	20 (11.0)	23 (12.0)	0.05	15 (12.6)	13 (10.9)	0.03
Living alone	140 (77.3)	172 (89.6)	0.33	98 (82.4)	102 (85.7)	0.09
ASM/height^2^, mean (SD), kg/m^2^	5.89 (1.15)	6.12 (1.21)	0.20	6.04 (1.21)	5.98 (1.16)	0.05
Grip strength, mean (SD), kg	16.82 (6.99)	20.21 (9.07)	0.42	17.45 (7.24)	17.87 (7.77)	0.06
Chronic conditions, mean (SD), No.	1.60 (1.06)	1.34 (1.04)	0.25	1.51 (1.06)	1.38 (1.06)	0.13
CES-D score, mean (SD)	9.54 (9.49)	9.54 (9.78)	0.00	9.76 (9.25)	9.87 (9.72)	0.01
MMSE-DS score, mean (SD)	24.21 (3.97)	24.59 (4.64)	0.09	24.23 (3.99)	23.76 (5.10)	0.10
Gait speed, mean (SD), m/s	0.65 (0.24)	0.74 (0.28)	0.36	0.66 (0.25)	0.68 (0.24)	0.08
Frailty phenotype, mean (SD)	2.21 (1.17)	1.80 (1.20)	0.30	2.20 (1.14)	2.09 (1.13)	0.10
Frailty Index, mean (SD)	0.27 (0.10)	0.23 (0.11)	0.33	0.26 (0.10)	0.25 (0.12)	0.03
Medical cost 6 mo pre-enrollment, mean (SD), $	714 (1732)	608 (1417)	0.07	543 (1144)	603 (1498)	0.05
Medical cost 1 y pre-enrollment, mean (SD), $	1349 (2317)	1206 (1959)	0.07	1143 (1869)	1127 (1819)	0.01

Abbreviations: ASM, appendicular skeletal mass; CES-D, Center for Epidemiologic Studies Depression; MMSE-DS, Mini-Mental State Examination for Dementia Screening; SMD, standardized mean difference.

### Intervention Costs and Intervention Adherence

All 119 participants were included in the group exercise and nutrition programs, 16 participants (13.5%) were enrolled in depression management, 59 participants (49.6%) were enrolled in polypharmacy management, and 86 participants (72.3%) were enrolled in the home hazard reduction program. The overall intervention adherence was 86.6%. The overall mean (SD) cost to provide the multicomponent intervention was $872 ($148) per participant. The per-participant mean (SD) cost of each intervention component varied, with exercise at $260 ($55); nutrition, $535 ($79); depression management, $106 ($16); polypharmacy management, $106 ($17), and home hazard reduction, $16 ($2). Detailed cost calculation results and adherence are presented in eAppendix 7 and eTable 4 in Supplement 1.

### All-Cause Mortality and LTCI Eligibility

**Figure 1.**
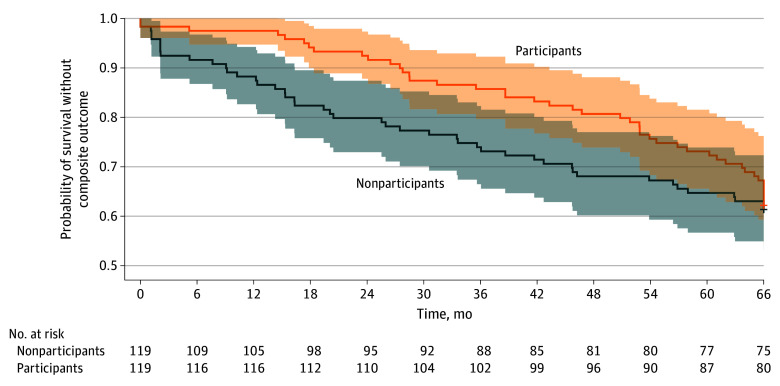
Probability of Survival Without Death and Long-Term Care Insurance Eligibility ^a^Restricted mean survival time difference, 2.86 months at 30 months (*P* = .003). ^b^Restricted mean survival time difference, 6.53 months at 66 months (*P* = .01).

The incidence of the composite outcome (all-cause mortality or LTCI eligibility) was 15 participants (12.6%) vs 27 nonparticipants (22.7%) at 30 months, and 39 participants (32.8%) vs 44 nonparticipants (37.0%) at 66 months. Kaplan-Meier curves for the composite outcome are shown in Figure 1. Participants had significantly longer survival free from death or LTCI eligibility, with RMST differences of 2.86 months at 30 months (28.53 vs 25.67 months; *P* = .003) and 6.53 months at 66 months (57.16 vs 50.63 months; *P* = .01) (eTable 5 in Supplement 1). The *E*-values for the composite risk at 30 and 66 months were 3.00 and 1.51, respectively (eTable 6 in Supplement 1). Incidence data are detailed in eTable 7 in Supplement 1, and analyses for mortality and LTCI eligibility separately are presented in eFigure 4 and eTable 8 in Supplement 1.

### Health Service–Use Costs

**Figure 2.**
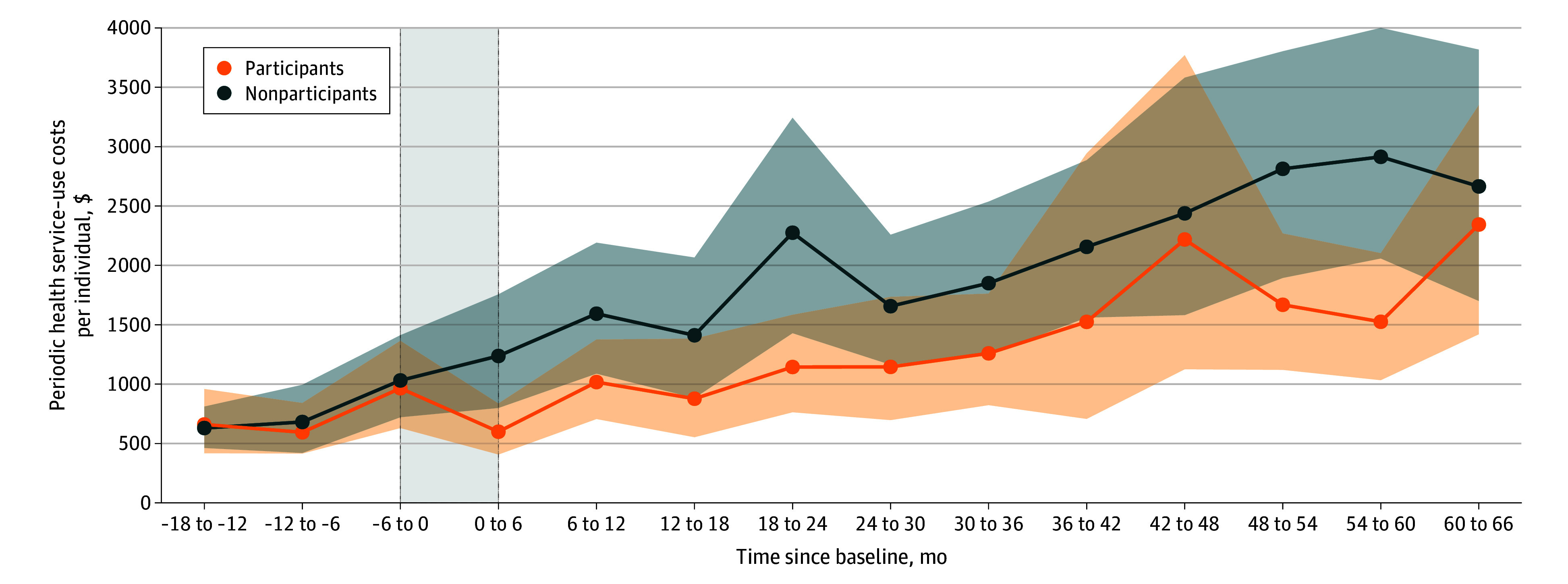
Trends in Periodic Health Service–Use Costs Per Individual for Participants and Nonparticipants Dotted lines indicate the intervention periods.

Figure 2 presents the per-person health service–use costs over 66 months. Prior to the intervention, costs were comparable between groups. During the active intervention phase (0-6 months) and thereafter, costs in the nonparticipants group steadily increased, whereas the participants group maintained consistently lower costs throughout follow-up. The between-group difference at 6 months ranged from $219 to $1389 per person after the intervention. Detailed breakdowns of medical and LTC costs are available in eTable 9 in Supplement 1. and the medians and IQRs of health service use costs are available in eTable 10 in Supplement 1.

**Figure 3.**
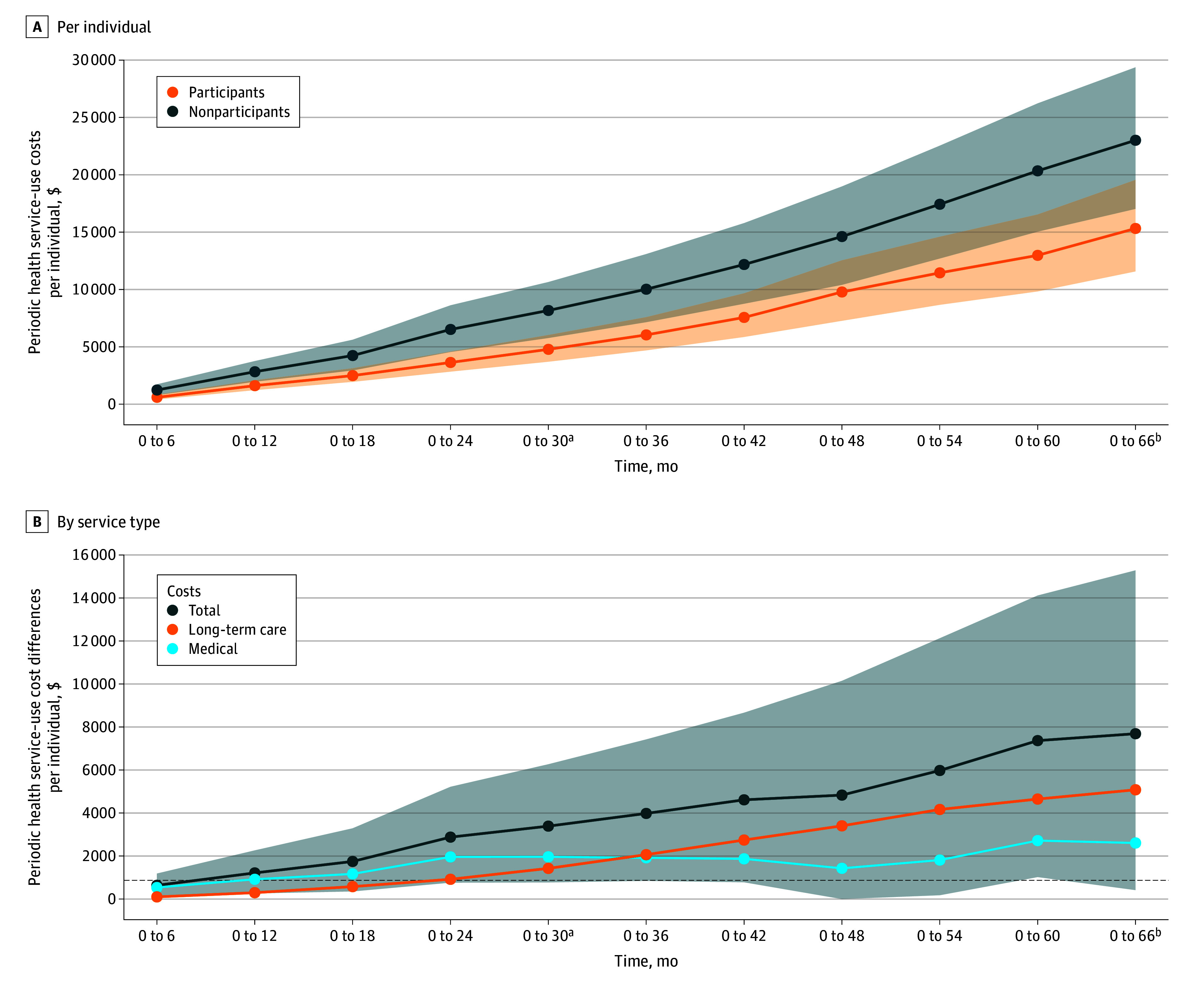
Cumulative Health Service-Use Costs and Cost Differences Per Individual Shaded areas indicate the 95% CIs. The dotted line indicates the intervention cost ($872). ^a^Difference: *P* = .008 at 30 months. ^b^Differenc: *P* = .01 at 66 months.

As detailed in Figure 3A, participants had increased cumulative savings in health care costs, statistically significant at both 30 and 66 months. By 30 months, the mean savings was $3390 (95% CI, $868-$5935) per person (*P* = .008), increasing to $7688 (95% CI, $1197-$14 615) by 66 months (*P* = .01). With an intervention cost of $872 per participant, the cost:benefit ratio was 3.89 at 30 months and 8.82 at 66 months (Figure 3B). Initially, most savings were attributable to reduced medical costs (57.9%), but by 66 months, reductions in LTC costs accounted for a greater share (66.1%). Further details are provided in eTable 11 and eTable 12 in Supplement 1, and inflation-adjusted results are in eTable 13 in Supplement 1. *E*-values for being in the top 20th percentile of health care costs were 1.56 at 30 months and 2.50 at 66 months (eTable 6 in Supplement 1).

### Multivariable-Adjusted and Subgroup-Specific Outcomes

Lower health service–use costs in the participants group remained evident after multivariable adjustment for age, gender, frailty index, and prior medical costs (eTable 14 in Supplement 1). In an exploratory subgroup analysis, greater cost differences were observed for females, individuals aged 80 years or older, those with a frailty index of 0.3 or higher, and those with higher prior-year medical costs compared with their respective counterparts (eTable 14 in Supplement 1).

### Sensitivity Analysis

The baseline characteristics after applying PSM with alternative calipers width of 0.10 and 0.15 SD, as well as inverse probability weighting are detailed in eTable 15 in Supplement 1. The risk ratios for the composite outcome (eTable 16 in Supplement 1) and the differences in cumulative health service use costs (eTable 17 in Supplement 1) remained consistent regardless of the analytical method used.

### Pattern of Health Service Utilization by Service Type and Principal Diagnosis

**Figure 4.**
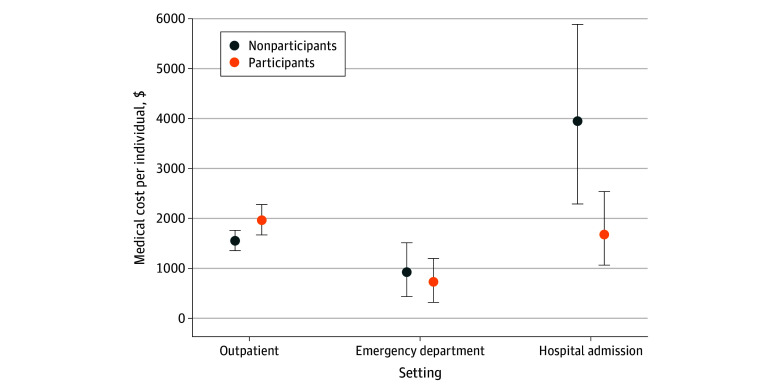
Mean Medical Costs Per Individual by Service Type for Participants and Nonparticipants

By 30 months, participants incurred higher outpatient care costs but lower hospital admission costs than nonparticipants (Figure 4; eTable 18 in Supplement 1). Analysis by principal *International Statistical Classification of Diseases and Related Health Problems, Tenth Revision* (*ICD-10*) categories found that cost savings were mainly driven by reduced utilization for circulatory (I00-I99), nervous system (G00-G99), and injury-related (S00-T98) conditions (eFigure 5 and eTable 19 in Supplement 1).

## Discussion

In this economic evaluation using data from a nonrandomized clinical trial, we found that participants in a 24-week multicomponent frailty intervention for socioeconomically vulnerable, community-dwelling older adults had longer survival without the composite outcome of mortality and LTCI eligibility over 66 months of follow-up compared with nonparticipants. Participants also had lower cumulative health service use costs, with savings of $3390 and $7688 at 30 months and 66 months, respectively, per individual, primarily attributable to decreased hospital admissions and lower LTC expenses. When considering the intervention cost of $872 per individual, the cost:benefit ratio was 3.89 at 30 months and 8.82 at 66 months, suggesting high return on investment for this multicomponent frailty intervention.

Building on our previous findings,^16,17,18^ this study found that the outcomes associated with this 24-week multicomponent program persisted up to 66 months, with cost-benefit advantages. These robust results may stem from our frailty-centered individualized approach. Unlike exercise- and nutrition-only interventions, our program included mental health, medication optimization, and home hazard reduction based on a comprehensive assessment to identify frailty-associated barriers that may interfere with intervention effectiveness.^2,4^ Furthermore, we addressed challenges such as adherence barriers and limited resources through collaboration with local public health centers and community partners.

Cost savings evolved over time: medical service reductions drove savings in the first 30 months, while LTCI-related savings became more prominent by 66 months. Although participants used more outpatient care, this was offset by reduced inpatient and LTC use, resulting in overall cost savings. This pattern aligns with earlier evidence that frailty-targeted interventions can reduce overall health care costs by lowering hospitalization rates.^14,26^ Notably, much of the cost reduction was driven by decreased utilization of services related to circulatory (*ICD-10* codes I00-I99) and injury-related conditions (*ICD-10* codes S00-T98).

Our study provides 2 key implementation insights. First, our results suggest that frail, older individuals experienced greater reductions in health service–use costs. Consistent with previous studies showing that frailty-based interventions are more cost-effective and improve quality of life in individuals with severe frailty,^12^ our findings underscore the value of prioritizing this group. Second, the optimal reintervention interval may be 2 to 3 years. While evidence remains limited, previous studies suggest that benefits of multicomponent interventions peak soon after implementation and gradually decline,^8,18^ with effects of a 2-year intervention sustained up to 7 years but not 11 years.^27^ In our study, the observed differences in mortality, LTCI eligibility, and health service use costs all narrowed at approximately 30 months. Given similar convergence patterns in disability and frailty,^17,18^ our findings suggest that reassessment and re-engagement at 2- to 3-year intervals may be appropriate for maintaining long-term benefits.

### Limitations

This study has inherent limitations as a nonrandomized trial, which we attempted to mitigate through several methodological approaches. Given the practical difficulties of conducting randomized clinical trials in rural older adult populations,^28,29^ our control group included eligible individuals who declined participation. We addressed this potential for selection bias by using PSM to balance key observed covariates. The preintervention health service use costs were similar between groups, supporting the validity of the match. To assess robustness to unmeasured confounding, we calculated *E*-values for our main outcomes (range, 1.5-3.0), indicating that moderate unmeasured confounders would be needed to explain away the observed effects.

The generalizability of this study is subject to 2 key considerations. First, the findings apply only to the participants included in the PSM, as unmatched individuals were excluded. To address this, we compared the characteristics of matched and unmatched populations and performed sensitivity analyses using alternative approaches. Second, the findings may not be generalizable to settings outside Pyeongchang, South Korea, although our prior work suggests the ASPRA cohort reflects the broader population of older adults in Korea.^19^

There are 2 other limitations to consider in interpretation. The relatively small sample size limits statistical power for detailed analyses, particularly for health care utilization by disease category. Accordingly, these findings should be considered exploratory rather than definitive. Furthermore, as this was a post hoc analysis, the possibility of type I error from multiple comparison exists. To minimize this concern, we restricted formal hypothesis testing to 1 health and 1 cost outcome at 2 landmark time points (30 and 66 months).

## Conclusions

In this economic evaluation of a nonrandomized trial involving community-dwelling older adults with mild to moderate frailty, a 24-week multicomponent intervention was associated with longer survival free from death or LTCI eligibility for up to 66 months. The intervention was also associated with cost savings, yielding per-person reductions in health service use costs equivalent to 3.89 and 8.82 times the initial program cost at 30 and 66 months, respectively. These savings were primarily driven by lower costs for long-term care and hospital admissions. These findings support the broader implementation of frailty-targeted interventions, particularly when formulating health care policies for aging populations in resource-limited settings.

## Notes

Supplement 1. eAppendix 1. Overview of the Aging Study of Pyeongchang Rural Area–Intervention Study (ASPRA-IS) and the present long-term analysis eAppendix 2. Details of the multicomponent intervention program eAppendix 3. Overview of the National Health Insurance Service (NHIS) and Long-Term Care Insurance (LTCI) in Korea eAppendix 4. Methods for calculating intervention costs and defining adherence eAppendix 5. Bootstrap analysis details eAppendix 6. Detailed specification for statistical analyses eAppendix 7. Results for calculating intervention costs and defining adherence eTable 1. Overview of the multicomponent intervention program eTable 2. List of deficit items used for Comprehensive Geriatric Assessment-based Frailty Index eTable 3. Baseline characteristics before and after propensity score matching eTable 4. Adherence rates and costs analysis of multicomponent interventions: exercise, nutrition, depression, polypharmacy, and home hazards eTable 5. Results of composite outcome (death or long-term care insurance eligibility) eTable 6. Relative risk and E-value sensitivity analysis eTable 7. Outcome incidence in the matched population eTable 8. Results of mortality and long-term care insurance eligibility eTable 9. Periodic health service-use costs per individuals throughout the observation period eTable 10. Median and interquartile range of periodic health service-use costs throughout the observation period eTable 11. Cumulative health service-use costs difference per individuals throughout the observation period eTable 12. Median and interquartile range of cumulative health service-use costs throughout the observation period eTable 13. Inflation-adjusted costs at 30 and 66 months and cost-benefit ratios eTable 14. Overall adjusted and subgroup analyses of the outcome on health service-use costs at 30 and 66 months eTable 15. Baseline characteristics by Matching Approach (Sensitivity Analyses) eTable 16. Incidence of composite outcome (mortality or long-term care eligibility) (Sensitivity analysis) eTable 17. Cumulative Health Service-Use Costs (US$) by Method (Sensitivity analysis) eTable 18. Pattern of Health Service-use Utilization by Service Type until 30 months eTable 19. Pattern of Health Service-use Utilization by Principal Diagnosis until 30 Months eFigure 1. Implementation and analytic framework of the ASPRA-Intervention Study eFigure 2. Study design flowchart eFigure 3. Distribution of propensity scores before and after matching eFigure 4. Probability of survival without death or long-term care insurance eligibility eFigure 5. Pattern of Health Service-use Utilization by Principal Diagnosis until 30 Months

Supplement 2. Data Sharing Statement
