# Errors in Figures 3 and 4

**DOI:** 10.1001/jamanetworkopen.2025.54082

**Published:** 2025-12-26

In the Original Investigation titled “Long-Term Health and Cost Outcomes of a 24-Week Multicomponent Frailty Intervention in Older Adults,”^1^ published November 12, 2025, the keys in Figure 3B and Figure 4 were mislabeled. This article has been corrected.^1^
