# Supplementary material for: Long-Term Health and Cost Outcomes of a 24-Week Multicomponent Frailty Intervention in Older Adults
Source: JAMA Netw Open. 2025 Nov 12;8(11):e2543278. doi: 10.1001/jamanetworkopen.2025.43278 (PMC12612941; doi:10.1001/jamanetworkopen.2025.43278)
Supplement: Supplement 1. — eAppendix 1. Overview of the Aging Study of Pyeongchang Rural Area–Intervention Study (ASPRA-IS) and the present long-term analysis eAppendix 2. Details of the multicomponent intervention program eAppendix 3. Overview of the National Health Insurance Service (NHIS) and Long-Term Care Insurance (LTCI) in Korea eAppendix 4. Methods for calculating intervention costs and defining adherence eAppendix 5. Bootstrap analysis details eAppendix 6. Detailed specification for statistical analyses eAppendix 7. Results for calculating intervention costs and defining adherence eTable 1. Overview of the multicomponent intervention program eTable 2. List of deficit items used for Comprehensive Geriatric Assessment-based Frailty Index eTable 3. Baseline characteristics before and after propensity score matching eTable 4. Adherence rates and costs analysis of multicomponent interventions: exercise, nutrition, depression, polypharmacy, and home hazards eTable 5. Results of composite outcome (death or long-term care insurance eligibility) eTable 6. Relative risk and E-value sensitivity analysis eTable 7. Outcome incidence in the matched population eTable 8. Results of mortality and long-term care insurance eligibility eTable 9. Periodic health service-use costs per individuals throughout the observation period eTable 10. Median and interquartile range of periodic health service-use costs throughout the observation period eTable 11. Cumulative health service-use costs difference per individuals throughout the observation period eTable 12. Median and interquartile range of cumulative health service-use costs throughout the observation period eTable 13. Inflation-adjusted costs at 30 and 66 months and cost-benefit ratios eTable 14. Overall adjusted and subgroup analyses of the outcome on health service-use costs at 30 and 66 months eTable 15. Baseline characteristics by Matching Approach (Sensitivity Analyses) eTable 16. Incidence of composite outcome (mortality or long-term care eligibil [file jamanetwopen-e2543278-s001.pdf]

Supplemental Online Content

Ji S, Lim J, An TJ, et al. Long-term health and cost outcomes of a 24-week multicomponent frailty intervention in older adults. *JAMA Netw Open*. 2025;8(11):e2543278.
doi:10.1001/jamanetworkopen.2025.43278

eAppendix 1. Overview of the Aging Study of Pyeongchang Rural Area–Intervention Study (ASPRA-IS) and the present long-term analysis
eAppendix 2. Details of the multicomponent intervention program
eAppendix 3. Overview of the National Health Insurance Service (NHIS) and Long-Term Care Insurance (LTCI) in Korea
eAppendix 4. Methods for calculating intervention costs and defining adherence
eAppendix 5. Bootstrap analysis details
eAppendix 6. Detailed specification for statistical analyses
eAppendix 7. Results for calculating intervention costs and defining adherence
eTable 1. Overview of the multicomponent intervention program
eTable 2. List of deficit items used for Comprehensive Geriatric Assessment-based Frailty Index
eTable 3. Baseline characteristics before and after propensity score matching
eTable 4. Adherence rates and costs analysis of multicomponent interventions: exercise, nutrition, depression, polypharmacy, and home hazards
eTable 5. Results of composite outcome (death or long-term care insurance eligibility)
eTable 6. Relative risk and E-value sensitivity analysis
eTable 7. Outcome incidence in the matched population
eTable 8. Results of mortality and long-term care insurance eligibility
eTable 9. Periodic health service-use costs per individuals throughout the observation period
eTable 10. Median and interquartile range of periodic health service-use costs throughout the observation period
eTable 11. Cumulative health service-use costs difference per individuals throughout the observation period
eTable 12. Median and interquartile range of cumulative health service-use costs throughout the observation period
eTable 13. Inflation-adjusted costs at 30 and 66 months and cost-benefit ratios
eTable 14. Overall adjusted and subgroup analyses of the outcome on health service-use costs at 30 and 66 months
eTable 15. Baseline characteristics by Matching Approach (Sensitivity Analyses)
eTable 16. Incidence of composite outcome (mortality or long-term care eligibility) (Sensitivity analysis)
eTable 17. Cumulative Health Service-Use Costs (US$) by Method (Sensitivity analysis)
eTable 18. Pattern of Health Service-use Utilization by Service Type until 30 months
eTable 19. Pattern of Health Service-use Utilization by Principal Diagnosis until 30 Months
eFigure 1. Implementation and analytic framework of the ASPRA-Intervention Study
eFigure 2. Study design flowchart
eFigure 3. Distribution of propensity scores before and after matching
eFigure 4. Probability of survival without death or long-term care insurance eligibility
eFigure 5. Pattern of Health Service-use Utilization by Principal Diagnosis until 30 Months

This supplemental material has been provided by the authors to give readers additional information about their work.

© 2025 Ji S et al. *JAMA Network Open*.

eAppendix 1. Overview of the Aging Study of Pyeongchang Rural Area–Intervention Study (ASPRA-IS) and the present long-term analysis

The ASPRA-IS was a prospective, single-arm study implementing a 24-week multicomponent intervention for older adults in a rural Korean community, conducted from August 2015 to January 2017.[1] From its inception, the study was developed through collaboration between academic teams and the local public health center (PyeongChang Health Center and County Hospital) to address real-world challenges such as limited resources, barriers to adherence, and difficulties in long-term follow-up. The detailed protocol for the intervention has been published previously.[1]

The study was carried out in Pyeongchang County, located approximately 180 km east of Seoul. Participants were recruited from the **Aging Study of Pyeongchang Rural Area (ASPRA),[2]** a population-based prospective cohort of 1,267 adults aged ≥65 years, representing more than 90% of older adults living in the county. All participants provided written informed consent.

We estimated that at least 78 participants would be needed to detect a 1-point change in the SPPB score over 6 months, assuming the standard deviation of the change score to be 2.1 points and 10% loss to follow-up.[3,4] There were no planned interim analyses or stopping rules.

Eligibility criteria included:

1. Age ≥65 years;
2. Residence in one of the three study regions;
3. Living alone or receiving medical aid services;
4. Provision of written informed consent.

Exclusion criteria were:

1. Inability to walk 100 m;
2. Institutionalization in the past 6 months;
3. Diagnosis of end-stage heart failure, end-stage renal disease, or metastatic cancer;
4. Cognitive impairment (Mini-Mental State Examination score ≤18);
5. Plans to move out of the study area within 6 months.

Socioeconomically vulnerable older adults (living alone or receiving medical aid) were prioritized for inclusion after consultation with local public health officials and community representatives. The trial was intentionally designed as a **pragmatic, regionally staged designed-delay study**, a common approach in community-based interventions, to overcome resource limitations and concerns about random allocation.[5]

© 2025 Ji S et al. *JAMA Network Open*.

As shown in **eFigure 1A**, the intervention was first implemented in one region (Region A), while another region (Region B) temporarily served as a control for short-term evaluation to guide expansion. The program was then sequentially rolled out to Regions B and C, so there was no permanent "control town," and all regions ultimately received the intervention. Immediate results of these phases have been reported previously.[1]

For the **present long-term analysis (eFigure 1B)**, we compared participants and non-participants within each region rather than at the region level. Propensity score matching was applied to balance baseline characteristics between groups. Thus, the current findings reflect the long-term impact of individual participation in the intervention program.

eAppendix 2. Details of the multicomponent intervention program

The 24-week multicomponent intervention was sequentially implemented across three regions: region A (August 2015–January 2016), region B (February 2016–July 2016), and region C (August 2016–January 2017). Before the intervention, participants received standard care through monthly or as-needed visits to local public health centers for chronic disease management, without any of the intervention components.

During the intervention, participants received a program that included structured group exercise, nutritional supplementation, depression management, medication review, and home safety assessments (detailed in **eTable 1**). The intervention team comprised two part-time certified exercise instructors (working 15% of standard hours), three part-time physicians (5% of standard hours), and two nurses or social workers (30% of standard hours).

Group exercise sessions were held twice weekly for 60 minutes at community centers, led by licensed trainers. Each session included approximately 20 minutes of resistance training, balance exercises, and cardiovascular conditioning. Participants also received written instructions and were encouraged to maintain 60 minutes of daily physical activity independently.

Nutritional supplementation consisted of two 125-mL ready-to-drink packets daily (200 kcal, 24.5 g carbohydrate, 13 g protein, 5.6 g essential amino acids, and 7 g fat), donated by Maeil Dairies Co., Ltd (Seoul, Korea). Nutritional consultations with a licensed nutritionist were provided before and after the 24-week intervention.

Participants who screened positive for depression (Center for Epidemiologic Studies Depression Scale >20) underwent monthly evaluations by geriatricians or psychiatrists and received medication management or supportive counseling as indicated.[6] Those taking five or more prescription medications had monthly reviews with geriatricians to identify and discontinue potentially inappropriate drugs, guided by the 2012 Beers criteria.[7] Additionally, nurses and social workers conducted home visits to identify and address fall hazards using the CDC Home Fall Prevention Checklist.[8]

© 2025 Ji S et al. *JAMA Network Open*.

eAppendix 3. Overview of the National Health Insurance Service (NHIS) and Long-Term Care Insurance (LTCI) in Korea

National Health Insurance Service (NHIS)

The National Health Insurance Service (NHIS) is Korea's universal Health Service-use system that provides comprehensive medical coverage to all citizens and registered residents. It operates under a single-payer model, ensuring access to essential Health Service-use services while managing national Health Service-use expenditures. The NHIS covers a wide range of medical services—including inpatient and outpatient care, diagnostic tests, prescription medications, and preventive health screenings—with the exception of non-covered (uninsured) services. Through its extensive claims database, the NHIS also serves as a valuable resource for epidemiological and Health Service-use policy research.

In this study, health service use-costs includes medical and long-term care costs, including both out-of-pocket and NHIS-covered expenses: medical costs encompassed all NHIS-covered medical services, while LTC costs included facility-, home-, and community-based care.

The study was conducted from the perspective of the Korean healthcare system

Long-Term Care Insurance (LTCI)

The Long-Term Care Insurance (LTCI) program, managed by NHIS, was introduced in 2008 to support older adults and individuals with disabilities who require assistance with daily living activities. LTCI provides financial and service-based support for long-term care needs, including home-based care, community services, and institutional care such as nursing home admission.

LTCI Eligibility Assessment Process

The LTCI eligibility determination involves a standardized, multi-stage assessment process:

1. **Application Process**: Individuals aged 65+ or those under 65 with age-related conditions (e.g., dementia, Parkinson's disease) can apply for LTCI benefits through local NHIS offices.
2. **Assessment Components**:
    - Standardized 52-item questionnaire evaluating:
        - Physical function (ADLs and IADLs)
        - Cognitive function
        - Behavioral symptoms

© 2025 Ji S et al. *JAMA Network Open*.

        - Nursing care needs
        - Rehabilitation needs
    - Medical evaluation by a physician
    - In-home assessment by trained NHIS evaluators
3. **Grading System**: Based on assessment scores, applicants are classified into:
    - Grade 1: Most severe impairment (≥95 points)
    - Grade 2: Severe impairment (75-94 points)
    - Grade 3: Moderate impairment (60-74 points)
    - Grade 4: Mild-to-moderate impairment (51-59 points)
    - Grade 5: Mild impairment with dementia (45-50 points with cognitive impairment)
    - Cognitive Support Level: Early dementia symptoms (below 45 points but with cognitive impairment)
4. **Determination Committee**: Final eligibility decisions are made by a committee composed of Health Service-use professionals, social workers, and public officials.
5. **Reassessment**: Beneficiaries undergo reassessment every 1-2 years to evaluate continued eligibility and potential changes in care needs.
6. **Appeal Process**: Applicants who are denied eligibility or disagree with their assigned grade can appeal through an established review process.

© 2025 Ji S et al. *JAMA Network Open*.

eAppendix 4. Methods for calculating intervention costs and defining adherence

1. Study Population for Cost Calculation

The total intervention costs were assessed by calculating both operational and direct costs, adding the assumed unmeasured cost (20% additional cost). The operational expenses incurred in running the program as well as direct expenses for delivering specific interventions across five categories: exercise, nutrition, depression management, polypharmacy management, and fall prevention. Cost for calculations were based on 187 individuals who participated in the intervention program, while the results are presented for 119 individuals whose data were merged with the National Health Insurance Service (NHIS) database and included in the propensity score-matched population. All costs are presented in US dollars, converted at an exchange rate of 1,141 Korean Won per 1 US dollar, which was the mean exchange rate during the intervention period (2015–2017, according to Economic Statistics System, Bank of Korea).

2. Cost Calculation Methods

A. Operational Costs:

- Coordinator salaries and related expenses constituted the primary operational costs
- These costs were allocated to each intervention proportionally based on their direct costs relative to total direct costs
- Per-participant operational costs were calculated by dividing each intervention's operational costs by the number of participants, assuming equal distribution

B. Direct Costs by Intervention Type:

Exercise:

- Per-session cost = Total direct expenses ÷ Cumulative number of participant sessions
- Individual participant cost = Per-session cost × Number of attended sessions

Nutrition:

- Nutritional drink costs = Unit price × Individual consumption
- Dietitian labor costs were distributed equally among participants
- Individual participant cost = (Unit price × Supplements consumed) + Per-participant dietitian labor cost

Depression and Polypharmacy Management:

- Individual participant cost = Outpatient consultation fee × Number of sessions attended

Fall Prevention:

© 2025 Ji S et al. *JAMA Network Open*.

- Per-session cost = Total direct expenditure ÷ Number of sessions delivered
- Individual participant cost = Per-session cost × Number of sessions attended

C. Total Cost Calculation:

- For each intervention: Total cost = (Operational cost + Direct cost) × 1.2 (Assuming unmeasured costs as 20% of the sum)
- Final calculations included cost-per-eligible participant for each intervention and total cost-per-eligible participant
- The method of inflation-adjusted analyses is described in Appendix 6.

2. Adherence Rate Definitions

A. Exercise:

- Total sessions: 48 (twice weekly)
- Adherence rate = Sessions attended ÷ Total sessions

B. Nutrition:

- Total supplement packs: 336 (twice daily)
- Adherence rate = Packs consumed ÷ Total packs distributed

C. Depression and Polypharmacy Monitoring:

- Total visits: 6 (monthly)
- Adherence rate = Visits attended ÷ Total visits

D. Home Hazard Reduction:

- Sessions conducted before and after intervention
- Adherence rate = Sessions attended ÷ Total sessions

© 2025 Ji S et al. *JAMA Network Open*.

eAppendix 5. Bootstrap analysis details

We performed nonparametric bootstrap analyses (B = 1000 replications) for health service–use costs, given their skewed distribution, to obtain uncertainty estimates while preserving the matched structure.

1. **Group-specific mean costs**

- For each group (participants and non-participants), 95% confidence intervals (CIs) were estimated using nonparametric bootstrap resampling within each group.
- Point estimates of mean costs were calculated directly from the observed data without resampling.

2. **Between-group differences in mean costs**

- To account for the matched design, paired bootstrap resampling was performed at the pair level.
- For each bootstrap sample, the difference in mean costs (non-participants–participants) was computed.
- The 95% CI was derived from the 2.5th and 97.5th percentiles of the bootstrap distribution.
- Point estimates of between-group differences in mean costs were calculated directly from the observed data.
- Two-sided p-values were calculated as twice the proportion of bootstrap replicates with effect estimates in the opposite direction from the observed difference to determine if there is a significant difference between two matched groups (participants and non-participants).

3. **Reporting strategy**

- To reduce the risk of multiple testing, p-values are reported only for two landmark time points (30 and 66 months) when comparing cumulative health service-use costs.
- For all other time points, only point estimates and CIs are presented.

© 2025 Ji S et al. *JAMA Network Open*.

eAppendix 6. Detailed specification for statistical analyses

1. **E-value Calculation**[9]

    - We calculated E-values for the composite outcome of mortality or long-term care insurance (LTCI) eligibility at 30 and 66 months.
    - We also calculated E-values for the likelihood of being in the top 20th percentile of health service–use costs at 30 and 66 months within the matched cohort.

2. **Inflation-Adjusted Analyses**

    - To account for inflation during the study period (2015–2021), we used annual health consumer price index (CPI) values reported by Statistics Korea: 2015 (96.298), 2016 (97.247), 2017 (98.109), 2018 (98.058), 2019 (98.516), 2020 (100.00), and 2021 (99.92).
    - The average annual increase was approximately 0.6%. We applied this uniform adjustment to express cumulative health service-use costs and intervention costs at the final time points (30 and 66 months) in inflation-adjusted terms.

3. **Multivariable-Adjusted and Subgroup Analyses**

    - To estimate overall treatment outcomes while accounting for potential confounders, and to assess heterogeneity of effects, we conducted both multivariable-adjusted analyses and subgroup analyses.
    - All analyses were performed in the matched cohort using generalized linear mixed models (GLMMs) with a random intercept for each matched pair, specifying a Gamma distribution with a log link for cumulative health service-use costs at 30 and 66 months.
    - Covariates were included based on their established associations with functional outcomes and healthcare costs: age and sex (demographic factors), frailty index (health status), and prior-year medical costs (baseline healthcare utilization).
    - **Sequential GLMMs:**
      • *Model 1 (Crude):* participation only.
      • *Model 2:* participation + age + sex.
      • *Model 3 (Fully Adjusted):* treatment assignment + age + sex + frailty index + prior-year medical costs.
    - **Subgroup Analyses:**
      Separate GLMMs were fitted for each subgroup: sex (male vs female), age (≥80 vs <80 years), frailty index (≥0.3 vs <0.3), and prior-year medical costs (above vs below median). Each model included fixed effects for treatment, the subgroup variable, and their interaction (e.g., treatment × sex). Treatment effects were reported as cost ratios (CRs) with 95% confidence intervals (CIs).

4. **Sensitivity Analyses**

    - To evaluate the robustness of the findings, we conducted additional propensity score matching using alternative caliper widths (0.1 and 0.15), as well as inverse probability weighting (IPW), and compared results to those from the primary analysis (caliper = 0.2).

5. **Patterns of Health Service Utilization by Service Type and Principal Diagnosis**

    - We compared health care utilization and costs between groups up to 30 months, stratified by type of service (outpatient visits, emergency department visits, and hospitalizations).

    - Additional analyses examined utilization by principal diagnosis categories based on ICD codes in claims data.

6. **Statistical Software and Packages**

    - All analyses were conducted using R software, version 4.0.3 (R Foundation for Statistical Computing), between June 2024 and August 2025.

    - The following packages were used: *MatchIt* (for propensity score matching), *survival* and *survRM2* (for survival analyses), and custom bootstrap resampling methods (for cost analyses).

    - To account for multiple comparisons for the two primary health outcomes (RMST differences at 30 and 66 months) and the two primary cost outcomes (cumulative health service-use cost differences at 30 and 66 months), a Bonferroni-adjusted significance level of **$p < 0.025$** was used for each family of tests. All hypothesis tests were 2-sided.

© 2025 Ji S et al. *JAMA Network Open*.

eAppendix 7. Results for calculating intervention costs and defining adherence

Note:

Cost calculations were performed for all intervention program participants (n=187). However, results presented below are for the propensity score matched (PSM) participants (n=119), who were selected from among those successfully linked to the National Health Insurance Service (NHIS) database (n=181).

1. Direct Costs by Intervention Type:

Exercise:

- Per-session cost: $4.1 (Total direct expenses $30,815 ÷ 7,524 Cumulative number of participant sessions)
- Individual cost: $4.1 × Number of attended sessions
- Total direct costs: $30,815

Nutrition:

- Per-participant nutritional drink costs: Unit price $1.1 × Individual consumption
- Per-participant dietitian labor cost: $15.2 (Calculated as: Monthly salary $1,578 × 0.1 participation rate × 18 months total intervention period ÷ 187 participants)
- Individual cost = ($1.1 × Supplements consumed) + $15.2
- Total direct costs: $60,840 (Drinks: $58,001 + Dietitian: $2,840

Depression and Polypharmacy Management:

- Consultation fee: $12.3 per session (2015 Korean primary care initial consultation fee)
- Individual cost: $12.3 × Number of sessions attended
- Total direct costs: Depression $2,147; Polypharmacy $6,515

Fall Prevention:

- Per-session cost: $5.2 (Total expenditure $1,402 ÷ 272 Cumulative number of participant sessions)
- Individual cost = $5.2 × Number of sessions attended
- Total direct costs = $1,402

3. Operational Costs (Total: $35,320)

Exercise: $10,700 ($57.2 per participant)

© 2025 Ji S et al. *JAMA Network Open*.

Nutrition: $21,125 ($113 per participant)

Depression: $746 ($22.6 per participant)

Polypharmacy: $2,262 ($22.6 per participant)

Fall prevention: $487 ($3.3 per participant)

4. Total Cost Calculation:

- Formula: Total cost = (Operational cost + Direct cost) × 1.2
- Calculations for matched participants:

Individual costs based on adherence

Intervention costs summed across operational, direct, and estimated unmeasured costs

Final results presented in **eTable 4.**

© 2025 Ji S et al. *JAMA Network Open*.

eTable 1. Overview of the multicomponent intervention program

Focus	Description of intervention
Exercise	• Intervention: 60-min group exercise session led by licensed trainers focusing on the following types. The intensity started with low-intensity exercises and increased intensity every month<br>1. Resistance (20 min): squat, plank, side plank, straight leg raises<br>2. Balance (20 min): one-leg standing, shifting from side to side, heel-to-toe walk<br>3. Aerobic/endurance (20 min): step up and down, quick pace, dancing<br>4. The exercise trainer was given instructions not to exceed 60–70% of the maximal exercise capacity based on the perceived exertion scale<br>• Target: all participants<br>• Frequency: twice a week
Nutrition	• Intervention: administration of 125 ml commercial liquid formula containing 200 kcal of energy, 24.5 g carbohydrate, 13 g protein, 5.63 g essential amino acid, and 7 g fat<br>• Target: all participants<br>• Frequency: twice a day
Depression	• Intervention: evaluation by a geriatrician or a psychiatrist and administration of supportive psychotherapy or antidepressant medication as clinically indicated<br>• Target: participants with a CES-D score >20 points at baseline<br>• Frequency: monthly
Polypharmacy	• Intervention: medication review by a geriatrician, and dose reduction or discontinuation of potentially inappropriate medications according to the 2012 Beer's criteria<br>• Target: participants taking five prescription medications at baseline<br>• Frequency: monthly
Home hazards	• Intervention: evaluation of home environment by a visiting nurse and a social worker using the Home Fall Prevention Checklist by the Centers for Disease Control and Prevention and modification of the environment to eliminate any identified hazard<br>• Target: all participants with any identified home hazard at baseline<br>• Frequency: trimonthly

CES-D, center for epidemiologic studies depression

Source: Oh G, Lee H, Park CM, Jung H-W, Lee E, Jang I-Y, et al. Long-term effect of a 24-week multicomponent intervention on physical performance and frailty in community-dwelling older adults. Age and Ageing. 2021;50:2157-66.

eTable 2. List of deficit items used for Comprehensive Geriatric Assessment-based Frailty Index

Category	Deficit Items	
Medical comorbidities (14 items)	• Hypertension<br>• Diabetes<br>• Cancer (other than a minor skin cancer)<br>• Chronic lung disease<br>• Heart attack<br>• Congestive heart failure<br>• Angina	• Asthma<br>• Arthritis<br>• Stroke<br>• Kidney disease<br>• Dementia<br>• Falls<br>• Constipation/IBS
Self-reported functional status and disability (21 items)	• Dressing<br>• Washing face, hair, or toothbrushing<br>• Bathing or shower<br>• Feeding<br>• Getting in and out of bed<br>• Toileting<br>• Fecal and urinary continence<br>• Fatigue<br>• Resistance<br>• Ambulation<br>• Pain: Question from EQ-5D	• Using telephone<br>• Using transportation<br>• Shopping<br>• Preparing own meals<br>• Housework<br>• Taking own medications<br>• Managing money<br>• Grooming<br>• Doing laundry<br>• Going out short distance without using transportation
Physical performance (5 items)	• Low physical activity: International Physical Activity Questionnaire (IPAQ) – Short Form (below 20 percentile in KNHANES)<br>• Dominant handgrip strength: < 26 kg for men and < 18 kg for women<br>• SPPB score: balance < 3 (1point) repeated chair stand < 3 (1point) gait speed <3 (1point)	
Mood (3 items)	• Center for Epidemiologic Studies Depression Scale >20<br>• Exhaustion: Positive answer to either of the following statements: “I felt that everything I did was an effort” or “I could not get going.” Participants that answered with “a moderate amount of the time (3–4 days)", or “most of the time” to either of these questions were considered as exhaustion.<br>• Anxiety: Question from European Quality of Life-5 Dimensions (EQ-5D)	
Cognition (1 item)	• Mini- Mental State Examination Dementia Screening <24	
Nutritional status (1 item)	• Malnutrition: Mini Nutritional Assessment-Short Form score ≤11	
Polypharmacy (1 item)	• Number of medications ≥5	
Social	• Social Frailty score ≥2	

© 2025 Ji S et al. *JAMA Network Open.*

interaction (1 item)	

CHS, Cardiovascular Health Study; FRAIL, Fatigue, Resistance, Ambulation, Illness, and Loss of weight; KNHANES, Korea National Health and Nutrition Examination Survey; SPPB, Short Physical Performance Battery

Source: Oh G, Lee H, Park CM, Jung H-W, Lee E, Jang I-Y, et al. Long-term effect of a 24-week multicomponent intervention on physical performance and frailty in community-dwelling older adults. Age and Ageing. 2021;50:2157-66.

© 2025 Ji S et al. *JAMA Network Open*.

eTable 3. Baseline characteristics before and after propensity score matching

	Participants			Non-participants		
	**Unmatched (N=62)**	**Matched (N=119)**	**SMD**	**Unmatched (N=73)**	**Matched (N=119)**	**SMD**
**Age groups, n (%)**			0.411			0.86
**65-69**	1 (1.6)	12 (10.1)		29 (39.7)	10 (8.4)	
**70-74**	11 (17.7)	25 (21.0)		18 (24.7)	27 (22.7)	
**75-79**	29 (46.8)	44 (37.0)		15 (20.5)	49 (41.2)	
**80-84**	17 (27.4)	28 (23.5)		6 (8.2)	21 (17.6)	
**85-**	4 (6.5)	10 (8.4)		5 (6.8)	12 (10.1)	
**Female, n (%)**	51 (82.3)	90 (75.6)	0.16	42 (57.5)	87 (73.1)	0.33
**Enrolled year, n (%)**			0.35			0.67
2014	7 (11.3)	26 (21.8)		37 (50.7)	24 (20.2)	
2015	34 (54.8)	48 (40.3)		17 (23.3)	46 (38.7)	
2016	21 (33.9)	45 (37.8)		19 (26.0)	49 (41.2)	
**Medical aid, n (%)**	5 (8.1)	15 (12.6)	0.15	10 (13.7)	13 (10.9)	0.09
**Living alone, n (%)**	42 (67.7)	98 (82.4)	0.34	70 (95.9)	102 (85.7)	0.36
**ASM/height^2, kg/m^2, mean (SD)**	5.62 (0.98)	6.04 (1.21)	0.38	6.36 (1.25)	5.98 (1.16)	0.316
**Grip strength, kg, mean (SD)**	15.62 (6.35)	17.45 (7.24)	0.27	24.02 (9.77)	17.87 (7.77)	0.696
**No. chronic conditions, mean (SD)**	1.76 (1.04)	1.51 (1.06)	0.23	1.27 (1.00)	1.38 (1.06)	0.101
**CES-D score, mean (SD)**	9.10 (10.00)	9.76 (9.25)	0.07	9.01 (9.93)	9.87 (9.72)	0.09
**MMSE-DS score, mean (SD)**	24.18 (3.97)	24.23 (3.99)	0.01	25.93 (3.42)	23.76 (5.10)	0.50
**Gait speed, m/s, mean (SD)**	0.64 (0.22)	0.66 (0.25)	0.10	0.85 (0.30)	0.68 (0.24)	0.61
**Frailty phenotype, mean (SD)**	2.23 (1.23)	2.20 (1.14)	0.02	1.32 (1.17)	2.09 (1.13)	0.676
**Frailty index, mean (SD)**	0.28 (0.10)	0.26 (0.10)	0.25	0.20 (0.09)	0.25 (0.12)	0.57

© 2025 Ji S et al. *JAMA Network Open*.

**Medical cost 6 months pre-enrollment, US$ (mean, SD)**	1,042(2479)	543(1144)	0.26	608(1,417)	603(1498)	0.01
**Medical cost 1 year pre-enrollment, US$ (mean, SD)**	1,334(2196)	1,143(1869)	0.10	1,206(1,959)	1,127(1819)	0.01

Note: ASM, appendicular skeletal mass; CES-D, center for epidemiologic studies depression; SMD, standardized mean difference; SPPB, short physical performance battery; MMSE-DS, mini-mental state examination for dementia screening.

© 2025 Ji S et al. *JAMA Network Open*.

eTable 4. Adherence rates and costs analysis of multicomponent interventions: exercise, nutrition, depression, polypharmacy, and home hazards

	Exercise	Nutrition	Depression	Polypharmacy	Home hazards	Overall
Eligible participants, n (%)	119 (100%)	119 (100%)	16 (13.45%)	59 (49.58%)	86 (72.27%)	119 (100%)
Definition of adherence	Attendance to group exercise sessions	Proportion of supplements consumed	Attendance to monthly visits	Attendance to monthly visits	Correction of any home hazards	Mean adherence of eligible interventions
Adherence (%)	81.39	87.93	88.54	88.98	90.12	86.63
Total operational cost (US$)	6,809	14,245	361	1,335	281	23,031
Total direct cost (US$)	19,040	38,782	1,043	3,865	799	63,529
Total estimated unmeasured cost (US$)	5,170	10,605	281	1,040	216	17,312
Total cost (US$)	31,019	63,632	1,685	6,240	1,296	103,872
Cost-per eligible participants (US$) (mean, SD)	260 (55)	535 (79)	106 (16)	106 (17)	16 (2)	872 (148)

© 2025 Ji S et al. *JAMA Network Open*.

eTable 5. Results of composite outcome (death or long-term care insurance eligibility)

**Restricted Mean Survival Time Analysis for Composite Outcome (Death or Long-term Care Insurance Eligibility) at 30 and 66 Months**					
Outcome Measures	Participants Group	Non-participants Group	Between-Group Difference*	Ratio†	p-value**
30-Month Analysis					
RMST, months	28.53 (27.60–29.45)	25.67 (24.05–27.28)	2.86 (1.00–4.72)	1.11 (1.04–1.19)	0.003
RMSL, months	1.47 (0.55–2.40)	4.34 (2.72–5.95)		0.34 (0.16–0.71)	
66-Month Analysis					
RMST, months	57.16 (54.16–60.16)	50.63 (46.45–54.82)	6.53 (1.38–11.68)	1.13 (1.02–1.25)	0.013
RMTL, months	8.84 (5.84–11.84)	15.37 (11.18–19.55)		0.58 (0.37–0.89)	

Values are presented as estimate (95% confidence interval)

RMST = Restricted Mean Survival Time; RMTL = Restricted Mean Time Lost

*Between-group difference calculated as (Participants – Non-participants)

**Two-sided p values for the between-group difference in RMST

†Ratio calculated as (Participants/Non-participants)

© 2025 Ji S et al. *JAMA Network Open*.

eTable 6. Relative risk and E-value sensitivity analysis

Outcome	Timepoint	Events, n (Participants / Non-participants)	Relative Risk (RR)	95% CI	E-value	E-value for Lower CI Bound
**Composite Outcome**	30 Months	15 / 27	0.56	0.31–0.98	3.000	1.038
	66 Months	39 / 44	0.89	0.62–1.27	1.509	1.000
**High Health Service-use Costs***	30 Months	22 / 26	0.85	0.51–1.42	1.559	1.000
	66 Months	19 / 29	0.66	0.38–1.12	2.503	1.000

CI, Confidence Interval.

* Defined as cumulative health service-use costs above the 80th percentile.

Events (n) shows the number of individuals in each group who experienced the outcome. The total number of individuals was 119 in both the participant and non-participant groups for all analyses.

The **E-value** is the minimum strength of association that an unmeasured confounder would need to have with both the exposure and the outcome to fully explain away the observed relative risk.

© 2025 Ji S et al. *JAMA Network Open*.

eTable 7. Outcome incidence in the matched population

	(A) Composite outcome		(B) Mortality		(C) Long-term care insurance eligibility	
	Participants (n=119)	Non-participants (n=119)	Participants (n=119)	Non-participants (n=119)	Participants (n=119)	Non-participants (n=119)
30 months, n (%)	15 (12.6)	27 (22.7)	6 (5.0)	9 (7.6)	10 (8.4)	21 (17.6)
66 month , n (%)	39 (32.8)	44 (37.0)	16 (13.4)	19 (16.0)	28 (23.5)	33 (27.7)

© 2025 Ji S et al. *JAMA Network Open*.

eTable 8. Results of mortality and long-term care insurance eligibility

**(A) Restricted Mean Survival Time Analysis at 30 and 66 Months**				
Outcome Measures	Participants Group	Non-participants Group	Between-Group Difference*	Ratio†
30-Month Analysis				
RMST, months	29.31 (28.66–29.96)	28.69 (27.77–29.61)	0.62 (-0.51–1.74)	1.02 (0.98–1.06)
RMSL, months	0.69 (0.05–1.34)	1.31 (0.39–2.23)		0.53 (0.16–1.70)
66-Month Analysis				
RMST, months	62.27 (60.10–64.44)	60.40 (57.67–63.13)	1.87 (-1.62–5.36)	1.03 (0.97–1.09)
RMTL, months	3.73 (1.56–5.90)	5.60 (2.87–8.33)		0.67(0.31–1.42)
**(B) Restricted Mean Survival Time Analysis for Long-term Care Insurance Eligibility at 30 and 66 Months**				
Outcome Measures	Participants Group	Non-participants Group	Between-Group Difference*	Ratio†
30-Month Analysis				
RMST, months	29.07 (28.33–29.81)	26.48 (24.97–27.99)	2.59 (0.91–4.27)	1.10 (1.03–1.17)
RMSL, months	0.93 (0.19–1.67)	3.52 (2.02–5.03)		0.27 (0.11–0.65)
66-Month Analysis				
RMST, months	59.65 (57.06–62.23)	53.77 (49.80–57.73)	5.88 (1.15–10.61)	1.11 (1.02–1.21)
RMTL, months	6.35 (3.77–8.94)	12.23 (8.27–16.20)		0.52 (0.31–0.87)

Values are presented as estimate (95% confidence interval)

RMST = Restricted Mean Survival Time; RMTL = Restricted Mean Time Lost

*Between-group difference calculated as (Participants – Non-participants)

†Ratio calculated as (Participants/Non-participants)

© 2025 Ji S et al. *JAMA Network Open.*

eTable 9. Periodic health service-use costs per individuals throughout the observation Period

**(A) Periodic Total Health Service-use costs per Individuals**			
Months	Participants (US$) (95%CI)	Non-participants (US$) (95%CI)	Mean Difference (US$) (95%CI)
-18–-12'	660 (427–996)	630 (460–815)	-30 (-373–279)
-12–-6'	594 (415–854)	681 (428–1,000)	87 (-284–484)
-6–0'	965 (629–1,404)	1,031 (687–1,401)	66 (-387–495)
0–6	599 (414–839)	1,237 (791–1,734)	638 (133–1,201)
6–12	1,017 (702–1,396)	1,593 (1,016–2,224)	576 (-55–1,295)
12–18	877 (545–1,342)	1,411 (873–2,009)	533 (-150–1,244)
18–24	1,144 (761–1,607)	2,275 (1,410–3,288)	1,130 (200–2,149)
24–30	1,145 (705–1,737)	1,656 (1,110–2,213)	511 (-214–1,252)
30–36	1,259 (837–1,760)	1,850 (1,228–2,504)	591 (-92–1,298)
36–42	1,524 (723–3,088)	2,156 (1,502–2,820)	632 (-927–1,748)
42–48	2,218 (1,121–3,728)	2,438 (1,564–3,468)	219 (-1,750–1,668)
48–54	1,668 (1,066–2,263)	2,813 (1,953–3,808)	1,145 (-35–2,295)
54–60	1,525 (1,006–2,089)	2,914 (2,089–3,843)	1,389 (348–2,445)
60–66	2,344 (1,442–3,472)	2,665 (1,755–3,688)	321 (-1,186–1,724)
**(B) Periodic Medical Costs per Individuals**			
Months	Participants (US$) (95%CI)	Non-participants (US$) (95%CI)	Mean Difference (US$) (95%CI)
-18–-12'	620 (386–966)	534 (398–691)	-86 (-421–205)
-12–-6'	523 (356–771)	593 (357–894)	70 (-294–442)
-6–0'	839 (536–1,217)	929 (612–1,292)	91 (-332–516)
0–6	514 (357–730)	1,052 (657–1,498)	537 (74–1,059)
6–12	942 (634–1,298)	1,323 (810–1,915)	381 (-206–1,017)
12–18	822 (495–1,265)	1,072 (591–1,638)	250 (-390–892)
18–24	1,024 (686–1,438)	1,815 (1,010–2,845)	791 (-143–1,803)
24–30	1,010 (587–1,582)	1,014 (607–1,507)	4 (-596–604)
30–36	1,028 (669–1,445)	981 (538–1,556)	-46 (-655–568)
36–42	1,241 (486–2,786)	1,193 (722–1,757)	-48 (-1,579–942)
42–48	1,815 (795–3,290)	1,374 (634–2,403)	-441 (-2,374–997)
48–54	1,200 (745–1,659)	1,584 (885–2,579)	384 (-584–1,363)
54–60	813 (501–1,191)	1,718 (998–2,585)	905 (31–1,800)
60–66	1,610 (845–2,605)	1,499 (723–2,480)	-111 (-1,446–1,125)
**(C) Periodic Long-term Care Costs per Individuals**			
	Participants (US$) (95%CI)	Non-participants (US$)	Mean Difference

© 2025 Ji S et al. *JAMA Network Open.*

		(95%CI)	(US$) (95%CI)
-18–-12'	40 (0–91)	96 (0–208)	55 (-23–159)
-12–-6'	71 (12–159)	88 (0–194)	17 (-100–146)
-6–0'	128 (16–281)	108 (9–226)	-20 (-196–145)
0–6	85 (0–205)	186 (60–330)	101 (-66–278)
6–12	75 (0–180)	270 (88–492)	195 (-3–443)
12–18	55 (7–121)	339 (118–604)	283 (82–549)
18–24	121 (–280)	460 (216–756)	339 (63–630)
24–30	135 (17–299)	642 (302–985)	507 (157–908)
30–36	231 (55–463)	868 (465–1,301)	637 (206–1,110)
36–42	283 (97–531)	963 (540–1,438)	680 (205–1,192)
42–48	404 (152–717)	1,064 (619–1,536)	660 (152–1,219)
48–54	468 (189–821)	1,230 (756–1,798)	761 (202–1,394)
54–60	712 (359–1,130)	1,196 (721–1,744)	483 (-114–1,143)
60–66	734 (376–1,146)	1,166 (697–1,707)	432 (-192–1,097)

© 2025 Ji S et al. *JAMA Network Open*.

eTable 10. Median and interquartile range of periodic health service-use costs throughout the observation period

**Months**	**Participants (US$)**	**Q1**	**Q3**	**Non-participants**	**Q1**	**Q3**
-18–-12'	246	107	560	273	110	605
-12–-6'	241	94	499	253	110	434
-6–0'	354	154	735	328	196	843
0–6	283	158	520	269	109	784
6–12	396	188	891	371	186	823
12–18	362	136	672	272	106	874
18–24	327	138	816	392	170	1,285
24–30	286	104	656	336	134	997
30–36	328	187	916	352	161	1,220
36–42	341	107	831	409	166	1,461
42–48	323	96	961	351	134	1,071
48–54	339	71	902	340	110	2,593
54–60	329	90	820	443	145	3,198
60–66	374	52	1,272	356	130	2,017

© 2025 Ji S et al. *JAMA Network Open.*

eTable 11. Cumulative health service-use costs per individuals throughout the observation period

**(A) Cumulative Total Health Service-use costs per Individuals**			
Months	Participants (US$) (95%CI)	Non-participants (US$) (95%CI)	Mean Difference (US$) (95%CI)
0–6	599 (414–839)	1,237 (791–1,734)	638 (133–1,201)
0–12	1,615 (1,227–2,091)	2,830 (1,957–3,761)	1,215 (332–2,156)
0–18	2,493 (1,950–3,137)	4,241 (2,941–5,621)	1,748 (465–3,190)
0–24	3,637 (2,840–4,596)	6,516 (4,552–8,623)	2,879 (922–5,189)
0–30	4,782 (3,704–6,034)	8,172 (5,769–10,651)	3,390 (868–5,935)
0–36	6,041 (4,692–7,593)	10,022 (7,150–13,089)	3,981 (1,020–7,318)
0–42	7,564 (5,861–9,670)	12,178 (8,758–15,789)	4,614 (998–8,624)
0–48	9,782 (7,271–12,546)	14,616 (10,404–18,983)	4,833 (-33–9,962)
0–54	11,451 (8,664–14,602)	17,429 (12,701–22,540)	5,978 (210–11,765)
0–60	12,976 (9,831–16,542)	20,343 (15,040–26,226)	7,367 (1,033–13,796)
0–66	15,320 (11,577–19,558)	23,008 (17,025–29,370)	7,688 (1,197–14,615)
**(B) Cumulative Medical Costs per Individuals**			
Months	Participants (US$) (95%CI)	Non-participants (US$) (95%CI)	Mean Difference (US$) (95%CI)
0–6	514 (357–730)	1,052 (657–1,498)	537 (74–1,059)
0–12	1,456 (1,112–1,868)	2,375 (1,663–3,249)	919 (135–1,720)
0–18	2,278 (1,760–2,866)	3,447 (2,355–4,702)	1,169 (28–2,427)
0–24	3,301 (2,556–4,175)	5,261 (3,674–7,180)	1,960 (94–4,012)
0–30	4,311 (3,332–5,394)	6,275 (4,428–8,490)	1,964 (-188–4,375)
0–36	5,339 (4,221–6,670)	7,257 (5,083–9,887)	1,918 (-453–4,756)
0–42	6,580 (5,168–8,380)	8,450 (6,061–11,442)	1,870 (-1,103–5,051)
0–48	8,395 (6,264–10,930)	9,824 (6,838–13,794)	1,429 (-2,835–5,720)
0–54	9,595 (7,224–12,342)	11,407 (8,121–15,870)	1,813 (-3,142–6,552)
0–60	10,408 (7,911–13,351)	13,126 (9,396–18,047)	2,718 (-2,544–8,007)
0–66	12,018 (9,175–15,283)	14,625 (10,436–19,796)	2,607 (-3,049–8,108)
**(C) Cumulative Long-term Care Costs per Individuals**			
	Participants (US$) (95%CI)	Non-participants (US$) (95%CI)	Mean Difference (US$) (95%CI)
0–6	85 (0–205)	186 (60–330)	101 (-66–278)
0–12	159 (–382)	456 (161–786)	296 (-57–702)
0–18	215 (11–481)	794 (307–1,357)	579 (93–1,215)
0–24	336 (59–692)	1,255 (521–2,083)	919 (229–1,804)
0–30	470 (112–915)	1,896 (892–3,025)	1,426 (429–2,651)
0–36	701 (214–1,305)	2,765 (1,378–4,203)	2,064 (718–3,654)
0–42	984 (369–1,766)	3,728 (1,995–5,551)	2,744 (1,018–4,793)
0–48	1,388 (570–2,339)	4,792 (2,663–7,058)	3,404 (1,228–5,958)

© 2025 Ji S et al. *JAMA Network Open*.

0–54	1,856 (817–3,112)	6,021 (3,451–8,726)	4,165 (1,502–7,294)
0–60	2,568 (1,232–4,196)	7,217 (4,237–10,292)	4,649 (1,315–8,188)
0–66	3,302 (1,729–5,167)	8,383 (5,127–11,905)	5,081 (1,268–9,121)

© 2025 Ji S et al. *JAMA Network Open*.

eTable 12. Median and Interquartile Range of Cumulative Health Service-Use Costs Throughout the Observation Period

Months	Participants (US$)	Q1	Q3	Non-participants	Q1	Q3
0–6	283	162	514	269	109	762
0–12	782	368	1638	786	405	2533
0–18	1299	590	2874	1255	658	3866
0–24	1707	958	4004	2333	976	4964
0–30	2382	1469	5006	2971	1317	6649
0–36	3140	1774	6757	3724	1797	8729
0–42	3893	2113	9522	4302	2329	10693
0–48	4896	2397	12731	4881	2617	13735
0–54	5640	2893	13900	5847	3272	16264
0–60	6082	3146	14791	7335	3764	19407
0–66	6730	3733	18171	7664	4245	25162

© 2025 Ji S et al. *JAMA Network Open*.

eTable 13. Inflation-Adjusted Costs at 30 and 66 Months and Cost-Benefit Ratios

Months	Participants (US$)	Non-participants (US$)	Mean Difference (US$)	Intervention Costs	Cost-benefit ratio
0–30 (priced at month 30)	4,800	8,205	3,402	882	3.86
0–66 (priced at month 66)	15,482	23,270	7,783	898	8.67

Values converted to the price level at 30 and 66 months, respectively, using an average annual CPI increase of 0.6%.

© 2025 Ji S et al. *JAMA Network Open*.

eTable 14. Overall Adjusted and Subgroup Analyses of the Outcome on Health Service-use Costs at 30 and 66 Months

**Analysis Group**	**Level**	**Effect Estimate (Cost Ratio) at 30 months**	**95% Confidence Interval**	**Effect Estimate (Cost Ratio) at 66 months**	**95% Confidence Interval**
**(A) Adjusted Analyses**					
**Model 1 (Crude)**	All	0.73	0.55–0.97	0.74	0.57–0.96
**Model 2 (Age and Gender adjusted)**	All	0.76	0.58–0.99	0.76	0.59–0.98
**Model 3 (Full adjusted)***	All	0.76	0.58–0.99	0.77	0.60–0.98
**(B) Subgroup Analyses**					
**Gender**	Male	0.96	0.51–1.82	0.98	0.53–1.81
	Female	0.65	0.46–0.91	0.68	0.50–0.93
**Age Group**	< 80 years	0.81	0.58–1.14	0.86	0.63–1.19
	≥ 80 years	0.57	0.32–0.98	0.57	0.34–0.95
**Frailty Index**	< 0.3	0.89	0.58–1.20	0.95	0.69–1.30
	≥ 0.3	0.42	0.26–0.67	0.40	0.22–0.71
**Prior-year Medical Costs**	Below Mean ($1,135)	0.77	0.50–1.04	0.84	0.61–1.15
	Above Mean ($1,135)	0.61	0.26–0.96	0.56	0.31–1.01

* Fully adjusted model includes age, gender, frailty index, and prior-year medical costs.

© 2025 Ji S et al. *JAMA Network Open.*

eTable 15. Baseline Characteristics by Matching Approach (Sensitivity Analyses)

Variable	Caliper 0.2, main (n=119/119)	SMD	Caliper 0.15 (n=116/116)	SMD	Caliper 0.1 (n=116/116)	SMD	IPW (weighted n = 176/191)*	SMD
**Age group (%)**		0.17		0.19		0.11		0.04
**65–69 y**	10.1 vs 8.4		12.1 vs 11.2		8.8 vs 10.5		13.7 vs 14.9	
**70–74 y**	21.0 vs 22.7		22.4 vs 15.5		21.9 vs 19.3		20.6 vs 21.2	
**75–79 y**	37.0 vs 41.2		38.8 vs 41.4		42.1 vs 39.5		36.8 vs 36.0	
**80–84 y**	23.5 vs 17.6		18.1 vs 21.6		17.5 vs 20.2		19.5 vs 18.6	
**≥85 y**	8.4 vs 10.1		8.6 vs 10.3		9.6 vs 10.5		9.3 vs 9.4	
**Female (%)**	75.6 vs 73.1	0.06	72.4 vs 78.4	0.141	76.3 vs 72.8	0.08	74.5 vs 73.5	0.02
**No. of diseases, mean (SD)**	1.51 (1.06) vs 1.38 (1.06)	0.13	1.52 (1.08) vs 1.58 (1.09)	0.056	1.52 (1.08) vs 1.58 (1.09)	0.06	1.49 (1.08) vs 1.46 (1.07)	0.03
**Frailty index, mean (SD)**	0.26 (0.10) vs 0.25 (0.12)	0.03	0.25 (0.11) vs 0.27 (0.10)	0.18	0.25 (0.11) vs 0.27 (0.10)	0.18	0.25 (0.10) vs 0.25 (0.12)	0.02

Participants vs. Non-participants

*In the IPW analysis, effective sample sizes (ESS) were calculated to assess the stability of the weights. The overall ESS was 318, with 153 in the intervention group and 165 in the non-participants group, compared with the nominal sample size of 238.

© 2025 Ji S et al. *JAMA Network Open.*

eTable 16. Incidence of composite outcome (mortality or long-term care eligibility) (Sensitivity analysis)

Method	30-month Events (n, %)	RR (95% CI)	66-month Events (n, %)	RR (95% CI)
**Caliper 0.2 (Main)**	15/119 (12.6%) vs 27/119 (22.7%)	0.56 (0.31–0.99)	39/119 (32.8%) vs 44/119 (37.0%)	0.89 (0.63–1.26)
**Caliper 0.15**	19/116 (16.4%) vs 25/116 (21.6%)	0.76 (0.44–1.30)	39/116 (33.6%) vs 47/116 (40.5%)	0.83 (0.59–1.16)
**Caliper 0.1**	17/116 (14.7%) vs 26/116 (22.4%)	0.65 (0.38–1.14)	37/116 (31.9%) vs 43/116 (37.1%)	0.86 (0.60–1.23)
**IPW**	12.9% vs 19.8% (weighted %)	0.60 (0.33 1.10)	31.0% vs 36.0% (weighted %)	0.80 (0.50–1.27)

Participants vs. Non-participants

© 2025 Ji S et al. *JAMA Network Open*.

eTable 17. Cumulative Health Service-Use Costs (US$) by Method (Sensitivity analysis)

**Method**	**0 to 30 months**	**Difference**	**0 to 66 months**	**Difference**
**Caliper 0.2 (Main)**	4,782 (3,704–6,034) vs. 8,172 (5,769–10,651)	3,390 (922–6,248)	15,320 (11,577–19,558) vs. 23,008 (17,025–29,370)	7,688 (589–14,528)
**Caliper 0.15**	5,347 (4,168–6,739) vs. 8,165 (5,768–10,821)	2,818 (-8–5,880)	14,786 (11,168–18,873) vs. 23,179 (17,234–30,332)	8,393 (1,070–16,669)
**Caliper 0.1**	5,011 (3,879–6,354) vs. 8,947 (6,695–11,667)	3,935 (1,111–6,633)	15,173 (11,812–19,101) vs. 23,273 (17,467–29,795)	8,100 (965–15,495)
**IPW**	5,003 (4,063–5,963) vs. 7,787 (5,813–10,039)	2,785 (515–5,039)	14,927 (12,137 18,226) vs. 21,495 (16,630–26,714)	6,567 (833–12,833)

Values are mean per person with 95% CI; Difference = Non-participants - Participants

© 2025 Ji S et al. *JAMA Network Open.*

eTable 18. Pattern of Health Service-use Utilization by Service Type until 30 months

**(A) Outpatient**		
	Participants	Non-participants
Number of visits, mean (SD)	71.3(43.3)	66.2(50.6)
Cost per person, mean (95% CI)	1964(1670–2277)	1552(1354–1761)
Cost difference (95% CI)	-412 (-793–-53)	
**(B) Emergency Department**		
	Participants	Non-participants
Number of visits, mean (SD)	0.68(1.6)	0.64(1.0)
Cost per person, mean (95% CI)	730(320–1197)	924(435–1513)
Cost difference (95% CI)	194 (-443–907)	
**(C) Hospital Admission**		
	Participants	Non-participants
Number of visits, mean (SD)	1.1(2.3)	2.3(5.3)
Cost per person, mean (95% CI)	1677(1065–2539)	3948(2289–5886)
Cost difference (95% CI)	2,270 (419–4,531)	

© 2025 Ji S et al. *JAMA Network Open*.

eTable 19. Pattern of Health Service-use Utilization by Principal Diagnosis until 30 Months

**(A) Infectious and Parasitic Diseases**		
	Participants	Non-participants
Number of visits, mean (SD)	0.94(2.16)	1.04(2.38)
Cost per person, mean (95% CI)	122(40–224)	64(27–109)
Cost difference (95% CI)	-58 (-174–32)	
**(B) Neoplasms**		
	Participants	Non-participants
Number of visits, mean (SD)	0.83(3.73)	0.43(2.65)
Cost per person, mean (95% CI)	312(40–655)	431(7–1099)
Cost difference (95% CI)	119 (-435–891)	
**(C) Diseases of the Blood and Blood-forming Organs and Immune System**		
	Participants	Non-participants
Number of visits, mean (SD)	0.34(1.90)	0.07(0.43)
Cost per person, mean (95% CI)	4(1–8)	2(0–4)
Cost difference (95% CI)	-2 (-7–2)	
**(D) Endocrine, Nutritional and Metabolic Diseases**		
	Participants	Non-participants
Number of visits, mean (SD)	3.02(7.35)	3.34(6.53)
Cost per person, mean (95% CI)	146(82–224)	114(72–162)
Cost difference (95% CI)	-32 (-110–34)	
**(E) Diseases of the Nervous System**		
	Participants	Non-participants
Number of visits, mean (SD)	1.12(3.38)	1.29(4.43)
Cost per person, mean (95% CI)	35(11–69)	375(12–1088)
Cost difference (95% CI)	340 (-42–1,059)	
**(F) Diseases of the Eye and Adnexa**		
	Participants	Non-participants
Number of visits, mean (SD)	3.25(5.25)	3.26(5.13)
Cost per person, mean (95% CI)	246(159–343)	254(161–360)
Cost difference (95% CI)	8 (-123–140)	
**(G) Diseases of the Ear and Mastoid Process**		
	Participants	Non-participants
Number of visits, mean (SD)	1.57(8.99)	0.76(1.57)
Cost per person, mean (95% CI)	28(13–52)	26(15–40)
Cost difference (95% CI)	-2 (-21–12)	

© 2025 Ji S et al. *JAMA Network Open*.

**(H) Diseases of the Circulatory System**		
	Participants	Non-participants
Number of visits, mean (SD)	11.14(11.85)	12.76(13.74)
Cost per person, mean (95% CI)	703(414–1127)	1741(825–2834)
Cost difference (95% CI)	1,038 (7–2,274)	
**(I) Diseases of the Respiratory System**		
	Participants	Non-participants
Number of visits, mean (SD)	10.13(11.13)	7.36(9.64)
Cost per person, mean (95% CI)	205(119–319)	257(93–533)
Cost difference (95% CI)	52 (-164–330)	
**(J) Diseases of the Digestive System**		
	Participants	Non-participants
Number of visits, mean (SD)	8.30(10.37)	6.55(9.96)
Cost per person, mean (95% CI)	370(159–813)	459(189–872)
Cost difference (95% CI)	88 (-407–589)	
**(K) Diseases of the Skin and Subcutaneous Tissue**		
	Participants	Non-participants
Number of visits, mean (SD)	2.72(5.85)	2.30(4.08)
Cost per person, mean (95% CI)	33(20–53)	45(22–85)
Cost difference (95% CI)	12 (-18–53)	
**(L) Diseases of the Musculoskeletal System and Connective Tissue**		
	Participants	Non-participants
Number of visits, mean (SD)	17.76(19.40)	20.30(33.78)
Cost per person, mean (95% CI)	831(544–1161)	650(474–876)
Cost difference (95% CI)	-181 (-563–182)	
**(M) Diseases of the Genitourinary System**		
	Participants	Non-participants
Number of visits, mean (SD)	2.35(4.52)	2.15(6.45)
Cost per person, mean (95% CI)	248(106–435)	111(36–225)
Cost difference (95% CI)	-138 (-342–57)	
**(N) Symptoms, Signs and Abnormal Clinical and Laboratory Findings**		
	Participants	Non-participants
Number of visits, mean (SD)	3.35(5.40)	2.25(4.88)
Cost per person, mean (95% CI)	81(57–112)	74(44–106)
Cost difference (95% CI)	-7 (-49–41)	
**(O) Injury, Poisoning and External Causes**		
	Participants	Non-participants
Number of visits, mean (SD)	1.95(3.50)	2.15(3.72)

© 2025 Ji S et al. *JAMA Network Open*.

Cost per person, mean (95% CI)	339(123–635)	593(218–1117)
Cost difference (95% CI)	254 (-211–814)	
**(P) for Factors Influencing Health Status and Contact with Health Services**		
	Participants	Non-participants
Number of visits, mean (SD)	0.50(1.53)	0.29(0.98)
Cost per person, mean (95% CI)	91(9–235)	26(6–57)
Cost difference (95% CI)	-65 (-212–29)	

© 2025 Ji S et al. *JAMA Network Open*.

eFigure 1. Implementation and analytic framework of the ASPRA-Intervention Study

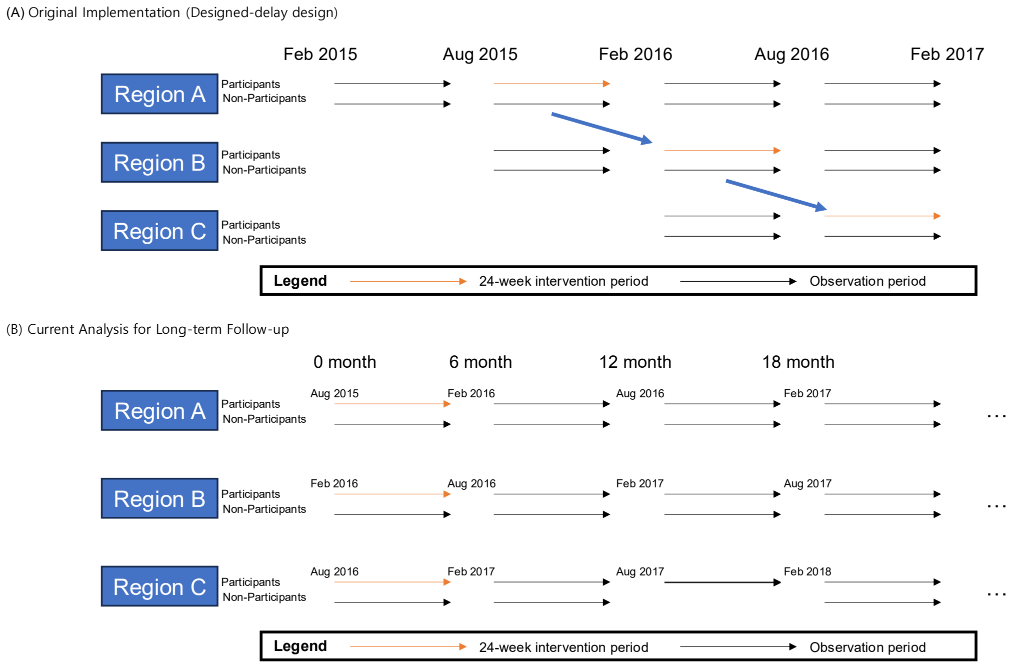

The intervention was sequentially expanded after completion in each region.

© 2025 Ji S et al. *JAMA Network Open*.

eFigure 2. Study Design Flowchart

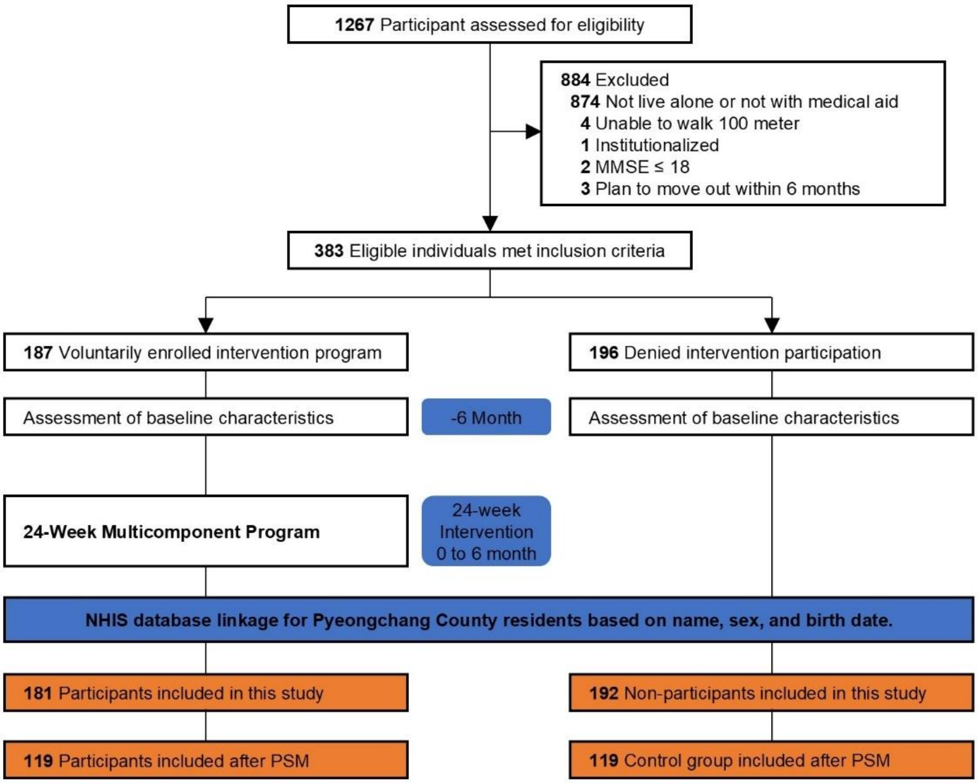

PSM, propensity score matching.

© 2025 Ji S et al. *JAMA Network Open*.

eFigure 3. Distribution of propensity scores before and after matching

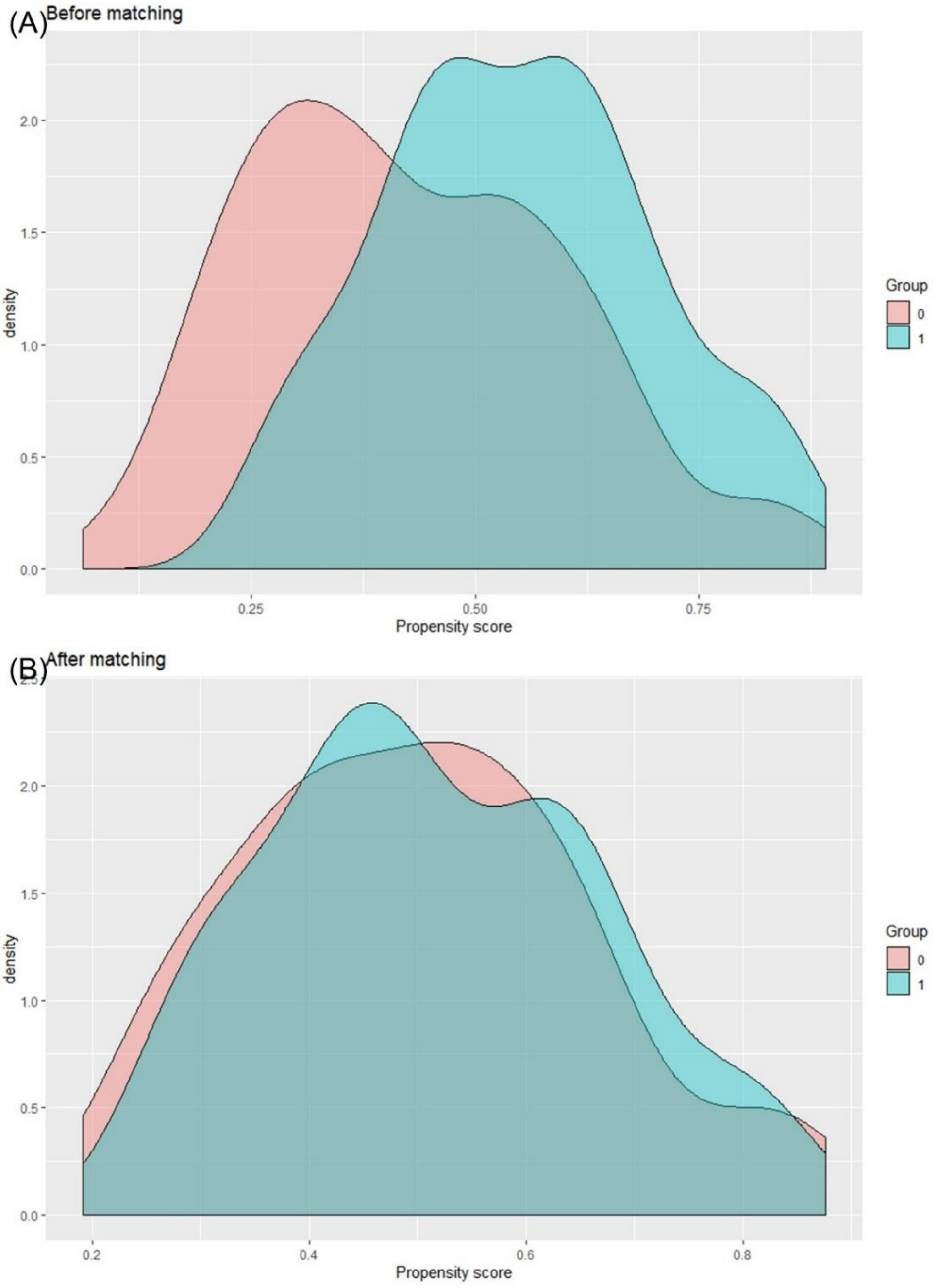

(A) Distribution before matching

(B) Distribution after 1:1 propensity score matching using a caliper of 0.2 standard deviations of the logit propensity score.

Kernel density plots of estimated propensity scores in participants (Group 1, blue) and non-participants (Group 0, red).

eFigure 4. Probability of survival without death or long-term care insurance eligibility

© 2025 Ji S et al. *JAMA Network Open*.

(A)

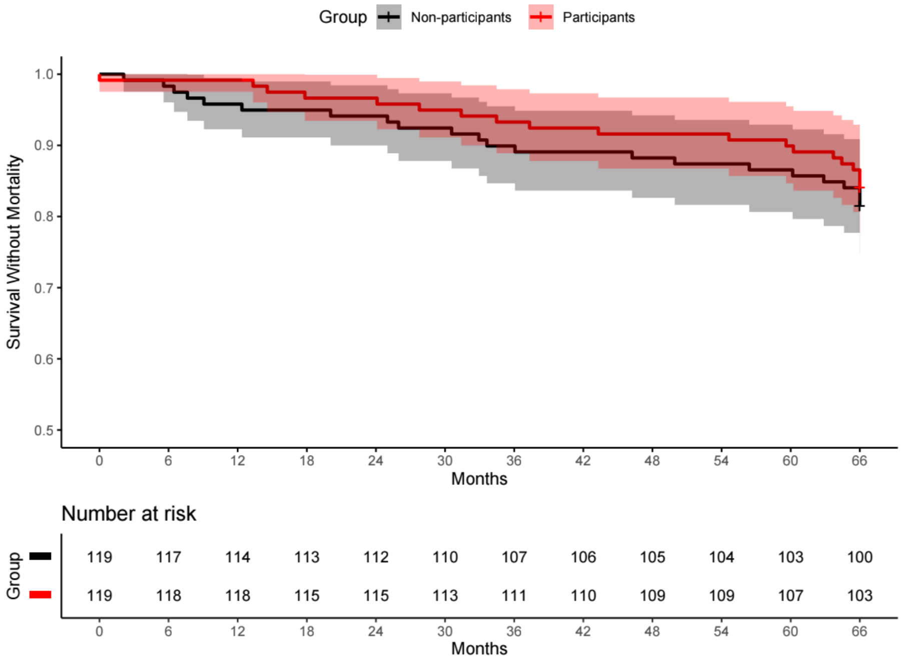

(B)

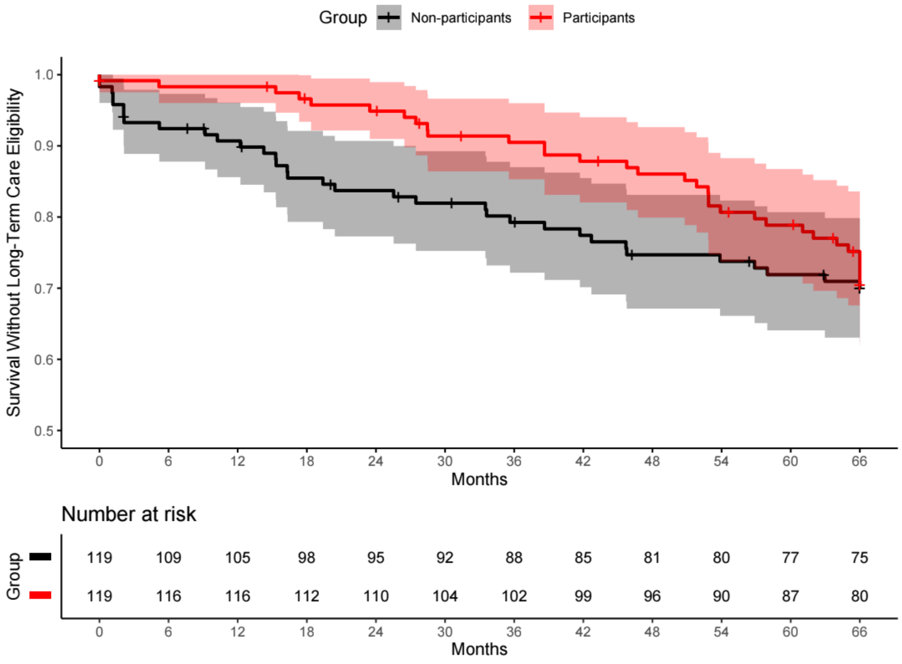

(A) Probability without mortality

(B) Probability without long-term care insurance eligibility

© 2025 Ji S et al. *JAMA Network Open*.

eFigure 5. Comparison of Health Service-use Utilization by Principal Diagnosis until 30 Months

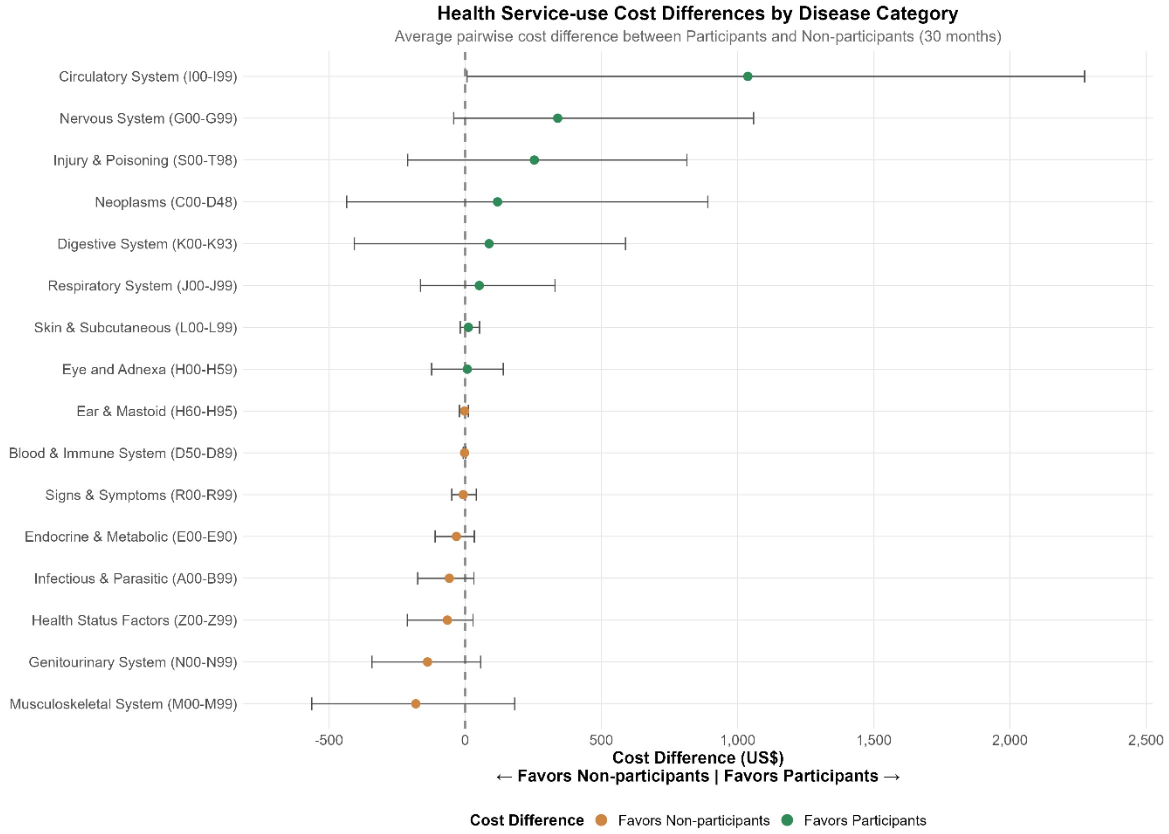

Error bars represent 95% confidence intervals

© 2025 Ji S et al. *JAMA Network Open*.

References

1. Jang IY, Jung HW, Park H, et al. A multicomponent frailty intervention for socioeconomically vulnerable older adults: a designed-delay study. *Clin Interv Aging*. 2018;13:1799-1814. doi:10.2147/cia.S177018
2. Jung HW, Jang IY, Lee YS, et al. Prevalence of Frailty and Aging-Related Health Conditions in Older Koreans in Rural Communities: a Cross-Sectional Analysis of the Aging Study of Pyeongchang Rural Area. *J Korean Med Sci*. Mar 2016;31(3):345-52. doi:10.3346/jkms.2016.31.3.345
3. Cameron ID, Fairhall N, Langron C, et al. A multifactorial interdisciplinary intervention reduces frailty in older people: randomized trial. *BMC Med*. Mar 11 2013;11:65. doi:10.1186/1741-7015-11-65
4. Kwon S, Perera S, Pahor M, et al. What is a meaningful change in physical performance? Findings from a clinical trial in older adults (the LIFE-P study). *J Nutr Health Aging*. Jun 2009;13(6):538-44. doi:10.1007/s12603-009-0104-z
5. Marshall DA, Willison DJ, Grootendorst P, et al. The effects of coxib formulary restrictions on analgesic use and cost: regional evidence from Canada. *Health Policy*. Nov 2007;84(1):1-13. doi:10.1016/j.healthpol.2007.04.010
6. Nguyen D, Vu CM. Current Depression Interventions for Older Adults: A Review of Service Delivery Approaches in Primary Care, Home-Based, and Community-Based Settings. *Current Translational Geriatrics and Experimental Gerontology Reports*. 2013/03/01 2013;2(1):37-44. doi:10.1007/s13670-012-0035-0
7. Bloomfield HE, Greer N, Linsky AM, et al. Deprescribing for Community-Dwelling Older Adults: a Systematic Review and Meta-analysis. *J Gen Intern Med*. Nov 2020;35(11):3323-3332. doi:10.1007/s11606-020-06089-2
8. Centers for Disease Control and Prevention (CDC). A Home Fall Prevention Checklist for Older Adults. . https://www.cdc.gov/steadi/pdf/check_for_safety_brochure-a.pdf
9. VanderWeele TJ, Ding P. Sensitivity Analysis in Observational Research: Introducing the E-Value. *Ann Intern Med*. Aug 15 2017;167(4):268-274. doi:10.7326/m16-2607

© 2025 Ji S et al. *JAMA Network Open*.
