# Supplementary material for: Long-Term Health and Cost Outcomes of a 24-Week Multicomponent Frailty Intervention in Older Adults
Source: JAMA Netw Open. 2025 Nov 12;8(11):e2543278. doi: 10.1001/jamanetworkopen.2025.43278 (PMC12612941; doi:10.1001/jamanetworkopen.2025.43278)
Supplement: Supplement 2. — Data Sharing Statement [file jamanetwopen-e2543278-s002.pdf]

Data Sharing Statement

Ji. Long-Term Outcomes of a 24-Week Multicomponent Frailty Intervention in Older Adults. *JAMA Netw Open*. Published November 12, 2025. doi:10.1001/jamanetworkopen.2025.43278

Data
Data available: Yes
Data types: Deidentified participant data, Other (please specify)
Additional Information: The dataset used in this study was created by linking data from the ASPRA cohort with the National Health Insurance Service (NHIS) database in Korea. Due to legal and institutional restrictions, the linked dataset is accessible only within the NHIS secure data analysis environment and cannot be publicly shared or transferred outside this setting. However, deidentified individual-level data from the ASPRA-IS dataset (prior to linkage with the NHIS), including a data dictionary and the statistical analysis plan, may be made available to qualified academic researchers upon reasonable request.
How to access data: Requests should be submitted to the corresponding author (IYJ) at onezero2@gmail.com any time after publication of this Article. All requests will be reviewed by the study team, and data access will require institutional approval and the execution of a data use agreement. Permitted uses include non-commercial academic research only. Commercial use is not permitted. No additional restrictions are placed on the types of statistical analyses.
When available: With publication

Supporting Documents
Document types: None

Additional Information
Who can access the data: Requests should be submitted to the corresponding author (IYJ) at onezero2@gmail.com any time after publication of this Article.
Types of analyses: Permitted uses include non-commercial academic research only. Commercial use is not permitted. No additional restrictions are placed on the types of statistical analyses.
Mechanisms of data availability: All requests will be reviewed by the study team, and data access will require institutional approval and the execution of a data use agreement.
